# Anthropogenically impacted lake catchments in Denmark reveal low microplastic pollution

**DOI:** 10.1007/s11356-022-19001-8

**Published:** 2022-02-19

**Authors:** Emilie M. F. Kallenbach, Nikolai Friberg, Amy Lusher, Dean Jacobsen, Rachel R. Hurley

**Affiliations:** 1 NIVA Denmark Water Research, Njalsgade 76, 2300 Copenhagen S, Denmark; 2 University of Copenhagen, Universitetsparken 4, Copenhagen Ø, Denmark; 3 NIVA, Økernveien 94, 0579 Oslo, Norway; 4 School of Geography, water@leeds, University of Leeds, Leeds, LS2 9JT UK; 5 Department of Biological Sciences, University of Bergen, 5020 Bergen, Norway

**Keywords:** Freshwater, Bivalves, Catchment, Microplastic sources, Monitoring, Bioindicator, Zebra mussel, Sediment

## Abstract

Microplastics have been detected in lake environments globally, including in remote regions. Agricultural and populated areas are known to congregate several inputs and release pathways for microplastic. This study investigated microplastic (50–5000 µm) contamination in five Danish freshwater lakes with catchments dominated by arable land use. The concentrations in sediments (n = 3/site) and the zebra mussel, *Dreissena polymorpha* (*n* = 30/site), were calculated and compared with catchment characteristics and environmental parameters. Microplastic concentrations in sediment were relatively low (average 0.028 ± 0.017 items/g dry weight sediment) whilst only a single microplastic was found in the mussels (average 0.067 ± 0.249 items/10 individual). Hence, no relationship between the number of observed microplastics in sediment and mussels could be identified, nor could a relationship between concentration in sediment and environmental parameters. As all lakes studied received their water from moderate to heavily anthropogenically impacted catchments, it was expected that they would be sinks for microplastic with high bioavailability. Based on the results of the present study, *D. polymorph*a were found to not be contaminated by microplastics in the five study lakes. Thus, our results suggest that these mussels do not interact with microplastics at low concentrations. We speculate that the results on sediment and biota could be explained by several factors related to regional differences in plastic use, species characteristics, sampling size, and the fact that finding no microplastic is not always reported in the scientific literature. Thus, the paper provides insight into the dynamics between the catchment, lake, and biota in systems with low microplastic concentration.

**Supplementary Information:**

The online version contains supplementary material available at 10.1007/s11356-022-19001-8.

## Introduction

Research increasingly indicates the significant role of freshwater systems in relation to microplastic pollution (Li et al. 2020). This in part refers to their capacity as a vector between terrestrial and marine environments, but also—and importantly—as vital ecological systems which can be affected by the potential impacts of microplastic contamination, following accumulation of particles and exposure in freshwater ecosystems (Horton et al. 2017b; Wagner and Lambert 2017). Indeed, microplastic has now been detected in sediment (e.g. He et al. 2020; Horton et al. 2017a; Lenaker et al. 2020; Rodrigues et al. 2018), in water (e.g. Deng et al. 2020; Earn et al. 2021; Nan et al. 2020; Simmerman and Coleman Wasik 2020), and in biota, including fish, birds, and invertebrates (e.g. Simmerman and Coleman Wasik 2020; Su et al. 2018; Windsor et al. 2019) of lakes, streams, and rivers.

Several studies highlight numerous potential sources and release pathways for microplastic to enter freshwater systems, e.g. road runoff, discharge from waste water treatment plants (WWTP), proximity to urban and industrial areas, and atmospheric deposition (Bergmann et al. 2019; Grbić et al. 2020; Horton et al. 2017a; Hurley et al. 2018a; Tibbetts et al. 2018; Xiong et al. 2018). There is a growing body of research establishing mass balance calculations for some microplastic sources (Boucher et al. 2019, Clayer et al. 2021, Prenner et al. 2021, Rasmussen et al. 2021); yet whilst most studies of microplastic pollution postulate some potential sources of microplastic, fewer studies have thus far quantified such releases. As a result, it is not yet known to what extent specific sources operate over different geographic regions, in response to different catchment characteristics, e.g. urbanisation, industry, agriculture, and recreational areas, or over different spatial and temporal scales, for a range of different freshwater environments. Furthermore, research elaborating the fate and transport of microplastics in the freshwater environment remains in its infancy, and relatively little is known about the combined influence of source dynamics and environmental processes that govern microplastic transport, deposition, and accumulation (Wagner et al. 2014). Research points towards the role floodplain and freshwater sediments have as accumulation zones for microplastic, particularly in lakes, but more research is needed to assess and describe the sources of microplastic to the freshwater environment (Clayer et al. 2021, Hurley et al. 2018a; Rodrigues et al. 2018; Turner et al. 2019; Xu et al. 2020). It is necessary to investigate different matrices to establish a baseline for the presence of microplastics across freshwater systems and assess the contribution from different sources.

Whilst monitoring microplastics in abiotic matrices (e.g. water and sediment) can provide a snapshot of environmental contamination, assessing the occurrence of microplastic in biotic samples reflects realistic exposures in these environments and suggests potential ecological impacts. Despite this, it is still largely unknown how the concentrations in one sample matrix could compare to another, for example, abiotic compared to biota samples (Bank and Hansson 2019; Ding et al. 2021; Li et al. 2018). For example, several methods exist for assessing bivalve species, and different methods have already been applied to freshwater bivalves which yielded conflicting results (Domogalla-Urbansky et al. 2019; Su et al. 2018). This adds to the discussion that the suitability of sample matrices must be assessed. Further research is needed to identify whether certain species are suitable for future monitoring activities.

It has been estimated that between 5,500 and 13,900 tons of microplastic could be released into the Danish environment every year (Lassen et al. 2015). Consequently, microplastics have been recorded in Danish marine waters, marine sediment, marine fish, wastewater treatment plants, and retention ponds, although, at present, no information on Danish freshwater ecosystems is available (Beer et al. 2018; Lassen et al. 2015; Lusher et al. 2021a; Simon et al. 2018; Vollertsen and Hansen 2017).

The purpose of this study was threefold: (i) to examine the ability of potential microplastic sources in the lake catchment to explain detected concentrations; (ii) to investigate the relationship between microplastic concentrations detected in sediments and *Dreissena polymorpha* at the same sites; and (iii) identify the most suitable indicator (abiotic/biotic) for the freshwater ecosystem investigated. It was hypothesised that lakes receiving their water from anthropogenic impacted catchments with several sources of microplastic would act as sinks and that sediment in these lakes would accumulate plastics. We defined anthropogenic impact based on catchment land use (% agriculture) and the presence of point source releases of sewage. Finally, we hypothesised that microplastic loads in the catchment should reflect catchment land use as well as associated, specific activities linked to inputs of microplastics, and these levels should be reflected in lake sediments and also mussels, if mussels reflect concentrations in the sediment and thus are suitable for use as bioindicators. Specifically, this study is the first one to report the presence or absence of microplastic in Danish freshwaters.

## Methods

### Field

#### Site selection strategy

Five lakes were selected from the national monitoring database for this study based on their catchment characteristics and record for environmental contamination as well as the presence of the mussel *Dreissena polymorpha* (see below). The five lakes are representative of Danish lakes, which are all shallow (the deepest Danish lake having a maximum depth of 37 m), lowland lakes (all below 100 m altitude), and with overlapping biological communities across relatively narrow environmental gradients (Søndergaard et al. 2020). Hence, the five lakes represent the most common Danish lake typology and provide a spatial variation in the samples, reducing in-lake as well as between-lake variability (Table 1). All lakes have an inflow and outflow, but retention time varies among the lakes (Table 1). Arresø (Lat 55.988668, Lon 12.083813), Hinge sø (Lat 56.257527, Lon 9.488302), Bryrup Langsø (Lat 56.020415, Lon 9.516921), and Ravn sø (Lat 56.105926, Lon 9.828211) are part of the national monitoring programme for water and nature, and thus environmental data are available for a range of parameters (Miljøstyrelsen 2017). The national monitoring programme for lakes (NOVANA) works to implement the EU Water Framework Directive, the Habitat Directive, and the Nitrate Directive. The programme design is based on the CIS guidelines from the Water Framework Directive on which parameters to be measured and the frequency of measurement. In general, physical/chemical and ecological parameters are included in both an operational monitoring programme—to help identify general trends—and a surveillance monitoring every second year—to document the environmental status (Miljøstyrelsen 2017).

**Table 1:** Study site characteristics for each lake, retention time in years, lake surface area in km^2^, volume of water (m^3^), catchment size (km^2^), and percentage of agriculture in the catchment

Lake	Retention time (years)	Lake surface area (km^2^)	Lake water volume (m^3^)	Catchment size (km^2^)	% catchment of agriculture
Arresø	3.1	39.54	1.23 × 10^8^	256.6	45.3
Bryrup Langsø	0.2	0.38	5.63 × 10^6^	49.9	71.7
Hinge sø	0.05	0.93	3.63 × 10^6^	53.8	83.8
Ravn sø	2.5	1.78	2.72 × 10^7^	57.2	72.3
Silkeborg Langsø	0.005–0.06	0.92	5.63 × 10^6^	1074.5	50.0

Specifically, Arresø was chosen because it is one of the most polluted lakes in Denmark. This has been linked in part to industrial activities and direct outflow of untreated wastewater until the late twentieth century (Mogensen 2005). Similarly, Silkeborg Langsø (Lat 56.174934, Lon 9.597014) was selected due to the presence of a wastewater treatment outflow close to the sampling site. All lakes have catchments dominated by agricultural land use, including fruit farms and vegetable fields but, predominately, cereals. In addition, populations of the invasive mussel *D. polymorpha* have been recorded in all five lakes. All lakes are used to a varying degree for recreation purposes such as swimming, fishing, and boating. Geographical characteristics for each lake are given in Table 1. Additional maps and pictures of the sampling sites can be found in the Supplementary Material (Figure SI1–SI6).

#### Sampling strategy and sample collection

Samples of sediment and biota were collected on the 14–15th of May 2019. The sampling period was chosen to be in spring before the summer stratification and bloom of cyanobacteria, reducing the food quality for *Dreissena polymorpha*, and, at the same time, the temperatures are higher than during winter, increasing the filtration rates. Full details of sampling time, sampling location, and biota size are provided in the Supplementary Material (Table SI1). The sediment compartment was targeted for sampling as it has been shown that the majority of microplastic particles sediment out from the water column in lake environments, including low-density microplastics which also settle out of suspension due to processes such as biofouling and aggregation (Corcoran et al. 2015; Rodrigues et al. 2018). Hence, concentrations of microplastic in the sediment are often higher than in the water phase, with the latter also being more laborious to sample (filtering of large volumes of water) and more prone to contamination of samples and high temporal variation. Rather, mussels were used as proxies of plastic content in the water compartment due to their filtering activity, which has been demonstrated in previous studies (Ding et al. 2021; Li et al. 2018) (see below for details on mussel sampling). At each site, a total of three sediment cores were collected using a Kajak sampler (*ø* = 5 cm, plexiglass) in the intertidal zone. Sediment cores were taken within 5 m of the mussel sampling location at each site and the replicates were taken randomly within 3 m distance. Maximum water depth at the coring location was 0.5 m. The top 5 cm of the corer (total length ~ 20 cm) was extruded with the use of an extruder rod, representing the surface sediments from the lake bed. Recent studies have shed light on the mobility of microplastics through the sediment (O'Connor et al. 2019), but the surface sediments were targeted here to represent recent deposition. The extruded sediments were transferred to a glass jar and sealed with tinfoil and a metal lid. The three cores taken at each site were pooled to produce a composite sample (294.5 cm^3^) to account for small-scale spatial variability in plastic content around the biota sampling site. As five different lakes were included, it was beyond the scope of the present study to address larger scale spatial variability within individual lakes. Temporal variability of plastic deposition was not included in the present study. Multiple studies have addressed seasonal and temporal variation in microplastic concentration (Iannilli et al. 2020; Rodrigues et al. 2018; Turner et al. 2019). Samples were stored at 5 °C for a maximum of 48 h before sample processing.

The invasive freshwater zebra mussel *Dreissena polymorpha* was selected as a study organism since they are often numerous, have high filtration rates, are mostly sessile, are easy to collect, and constitute an important organism in freshwater systems as they can change nutrient dynamics and species composition dramatically. A minimum of 30 mussels with a size between 1 and 3 cm were collected by hand or using a metal sieve from the shores or piers in each of the five lakes. The mussels were collected between 20 and 50 cm below the water surface. Only mussels attached to hard substrate were collected. The mussels were stored in zip-lock bags and frozen (− 20 °C) shortly after collection and until analysis.

### Microplastic analysis

#### Sediment

Wet sediment was first gently sieved through a 5 mm sieve to remove large debris such as stones and organic material. The > 5 mm fraction was discarded as it was above the size threshold for microplastics used in this study and no visible larger plastics were observed in this fraction when inspected under a stereoscope. Thereafter, the < 5 mm sediment was dried at 40 °C for 5 days. The containers were kept covered with aluminium foil for the duration of the drying, and the sediments were frequently manually stirred using a glass spatula to avoid clumping of the sediment into hard aggregates and to help homogenise the sample. Subsamples of 225 g (dry weight (d.w.)) sediment per site were transferred to 50-ml Falcon tubes. Falcon tubes were filled with ~ 20 mL of sediment and ~ 35–40 mL NaI (*ρ* = 1.8 g/cm^3^) for density separation. The tubes were sealed, shaken until all the material was thoroughly mixed, and left for 24 h to completely settle. The supernatant was sieved through a 50 µm sieve and the retained material was transferred into Erlenmeyer flasks. The density separation process was repeated a second time to ensure good recovery of particles, based on the findings of Hurley et al. (2018a). The retentate from the first density separation was found to contain organic material which could hinder subsequent visual and chemical analysis. Therefore, the material was treated with 30% H_2_O_2_ and incubated at 40 °C for 24 h to reduce the organic content (Hurley et al. 2018b). This method at the given concentration and reaction time has been showed to leave polymers unaffected; however, bleaching and degradation can happen when reaction time is prolonged (Hurley et al. 2018b; Karami et al. 2017; Nuelle et al. 2014). The digested material was then vacuum filtered onto 47 mm Whatman GF/A filters. The supernatant from the second density separation was also filtered onto additional GF/A filters. All filters were placed in individual petri-dishes, covered by a lid, and allowed to air-dry at room temperature ahead of the subsequent analysis.

#### Mussels

Mussels were analysed and soft tissue was digested in accordance with the method described by Bråte et al. (2018). The maximum shell length (mm) and width (mm) of all individuals were measured with a vernier caliper (KABI precision, accuracy 0.005 mm) and recorded to the nearest 0.1 mm. Thereafter, the mussels were opened, the soft tissue was rinsed with filtered water and loosened, and the byssus threads were removed. The soft tissue was weighed on an analytical balance (accuracy = 0.0001 g, Sartorius Research, R160D, Goettingen, Germany), transferred to conical flasks, and covered with aluminium foil. For each station, a composite sample of 10 individuals of approximately the same size (1–2.5 cm) was grouped in the conical flasks, with three replicates created for each site (150 individuals in total). Several studies now recommend the use of 10% KOH to digest soft tissues, based on the preservation of microplastics (Dehaut et al. 2016; Thiele et al. 2019). Approximately 20–50 mL of 10% KOH was added to each conical flask and incubated at 40 °C for 24 h with magnetic stirring, as this method has been shown to not degrade plastic polymers (Bråte et al. 2018; Pfeiffer and Fischer 2020). This corresponded to a ratio of 1:10 (wet weight, w.w.) between KOH solution and mussel tissue. Finally, the digested material was vacuum filtered onto silver membrane filters (pore size 0.5 µm, 13 mm diameter; Sterlitech, USA). As mussels were not dried prior to weighing and digestion, results are given as particles per g w.w.

#### Visual and chemical characterisation

Visual identification was used as an initial screening step to eliminate potential non-plastic particles prior to FTIR analysis. This was carried out in accordance to the method described by Lusher et al. (2020). All filters from the sediment samples were visually inspected for microplastics using a Nikon SMZ 745 T stereomicroscope at 20 × magnification. The lower size limit for visual analysis was 50 µm, as this represents the lowermost size at which it is possible to pick with tweezers. All suspected microplastics were photographed using an Infinity 1 camera, and the shape (e.g. fragment and fibres) and colour were recorded. The maximum and minimum Feret’s diameter was measured for all particles using the Infinity Analyze (v.6.5.4) software package, following calibration using a standard. Visual identification was used to eliminate potential non-plastics (according to Lusher et al. 2020) FTIR was then used to confirm or deny the results of the visual analysis. All suspected microplastics from the visual analysis step were individually transferred to a diamond compression cell (DC-3, PerkinElmer) and analysed for polymer type on a PerkinElmer Spotlight 400 µFTIR in transmission mode at the NIVA Microplastic Laboratory, Oslo, Norway. The nominal spectral resolution was 4 cm^−1^ across a spectral range of 4000–600 cm^−1^, and 2 co-scans were taken for each spectrum. Blank measurements were taken each time the diamond compression cell was loaded onto the FTIR, representing approximately every 1–4 particles. In total, 78 particles from the environmental samples and 42 particles from the blanks were analysed, representing all suspected particles from the visual identification step.

The silver membrane filters used for the mussels were analysed on a PerkinElmer Spotlight 200 µFTIR at the Cphbusiness Laboratory, Denmark. All particles were photographed through a series of optical images produced in the Spectrum10 software (v. 10.6.2.1159) connected to the instrument. All suspected microplastics were manually identified from these images, particle characteristics such as shape and colour were recorded, and ImageJ was used to measure particle size following the same procedure as for the sediment samples and using the internal scale provided by Spectrum10 for calibration. The lower size limit of detection was 50 µm for the mussel samples. Suspected microplastics were analysed in reflectance mode, at a spectral resolution of 4 cm^−1^, and within the spectral range 4000–600 cm^−1^. Background scans were made for each filter, in the first instance, but where there were more than 10 particles present on a filter, additional background scans were made for every 10 particles. All suspected microplastics (62 particles in total) were analysed with µFTIR, including 21 from the blank samples.

All FTIR spectra obtained for both the sediment and mussel samples were compared to the BASEMAN reference library (Primpke et al. 2018) on the base spectra. Additionally, comparison of the 1st and 2nd derivative of each spectrum and the reference spectra were made. All spectra were manually inspected and compared with reference spectra to confirm the polymeric composition. Hit quality indices derived from library search algorithms were consulted, using a tentative lower score of 0.6, but all spectra were also checked for the occurrence and positioning of characteristic peaks from reference spectra of potential interference and the common forms of contamination (e.g. moisture and biofilm residue) that can result in a lower match score.

### Quality assurance/quality control

Prior to sampling, all equipment and storage devices used for sampling in the field were washed thoroughly three times with filtered demineralised water (filtered using VacuCap 90 filters with 0.2 µm super membranes; Pall Corporation, Australia). All sample equipment was washed again with filtered demineralised water between sampling sites. No field blanks were carried out, due to thorough cleaning of all containers and the minimal time that the samples were exposed to air. This was coupled with thorough logging of all plastic types both in contact with the samples and in the vicinity of the sampling, allowing for control of contamination from these.

All water and reagents used for laboratory work were filtered prior to use following the same approach as for the filtered deionised water used in the field. All laboratory work was carried out in a fume hood which was cleaned twice with filtered demineralised water and once with filtered 76% ethanol prior to use. Nitrile gloves, 100% cotton/wool clothing, and 100% cotton laboratory coat were worn during laboratory work. All equipment was rinsed three times with filtered demineralised water prior to sample handling and between each sample to minimise contamination and cross contamination. A series of procedural blanks were carried out for both sediment and biota samples in the laboratory. Only fluids, and no sediment or biota, were added to these blanks, which were treated to an identical sample processing procedure as the environmental samples. Biota and sediment processing were carried out in separate batches. For each batch of samples, six blanks were carried out (therefore 12 in total across both sample types), and these were spaced out regularly between the real samples and processed at the same time. No recovery tests were performed on this sediment samples; however, the same method and procedure were applied on sediment analysed simultaneously and in the same laboratory by the same personal, by adding 10 PE beads and 10 polyester fibres. Recovery tests were performed for both sediment and mussels. Briefly, 10 polyethylene beads (400–450 µm) and 10 polyethylene terephthalate fibres (~ 300–600 µm) were added to falcon tubes containing freshwater sediments from Norway, and 10 tyre fragments (250 µm), 10 polyethylene beads (300 µm), and 10 polyethylene terephthalate fibres (~ 300–600 µm) were added to 5 samples containing mussel tissue. These were treated to an identical sample processing and analytical procedure as the real environmental samples. The discrepancy between particles used for the different matrices represent the availability of microplastic reference materials in the laboratories where the processing took place.

### Environmental data

Environmental data for the lakes was gathered to contextualise the microplastic data from the sediments and mussels and to test the hypothesis related to the relationship between environmental concentrations and catchment characteristics. These data do not provide exact measures of the actual sources of microplastic pollution but do act as proxies for potential sources.

Environmental data, total nitrogen (TN; mg/l) and total phosphorous (TP; mg/l), was extracted from the publicly available national monitoring database (www.odaforalle.au.dk). Average TN and TP concentrations from 2014, 2016, and 2018 were used for Hinge sø, Ravn sø, and Arresø whereas average concentrations from 2015, 2017, and 2019 were used for Bryrup Langsø, and the years 2011 and 2016 were included for Silkeborg Langsø. The variance in years for the different lakes is due to the design of the national monitoring programme, where not all lakes are monitored each year. Average concentrations across 2–3 years were used in order to smooth the influence of any annual variability and to account for microplastic loads accumulate over time. Data from the months May–September were used, representing the data availability. TN and TP were chosen as excess amounts originated from the same sources as potential microplastic pollution, namely sewage and agriculture. This contention is, for example, supported by Li et al. (2019) who found a correlation between sediment microplastic concentrations and TN. Excess concentrations of these nutrients in study lakes also verify a link between catchment land use and water quality with TN being primarily indicative of agricultural impact and TP a mix of discharge from WWTP, erosion, and runoff from soil (Li et al. 2019). In addition, yearly outflow from point sources covering wastewater effluent, industrial effluent, stormwater discharge, and discharge from scattered dwellings both as emissions directly into the five lakes, as well as into their catchments, was provided by the Danish Environmental Ministry. For each of these five outflow types, the chemical oxygen demand (COD) is given because it is used in the Danish environmental legislation to regulate sewage outflows and hence routinely measured (Miljøstyrelsen 1999). Overall, high concentrations of COD in recipient waters will lower oxygen concentration and negatively influence biota (Hu and Grasso 2005). The five outflow types were summed for the years 2017, 2018, and 2019 to reduce yearly variation and divided by the lake surface area, to account for dilution.

To characterise agricultural activity in the catchment, crop type was recorded by manually inspecting crop maps provided by OneSoil (https://map.onesoil.ai/) from the year 2018. These maps are based on satellite photos from sentinel-2, ground truth data, and use machine learning techniques to ascertain crop types. Spectral images from an entire season are combined to characterise crop type; however, some uncertainty will be associated with this. At a minimum, each field highlighted by the OneSoil maps was confirmed to be agricultural by manual verification of the satellite images ensuring that private orchards and gardens were not included, although exact crop type could not be verified further than the data available on OneSoil. To ascertain the potential plastic inputs associated with agricultural activity in the catchments, three crop types were identified as being potentially subject to agricultural plastic use (i.e. the use of thin films for mulching or tunnel/greenhouse systems): fruit (not including apples and pears), potatoes, and vegetables. It was not possible to confirm the use of agricultural plastics in these fields using the satellite images, as plastic may also be used under the ground, may be dark in colour, or may be only used during certain times of the year and therefore difficult to reliably detect from this data source. Nevertheless, agricultural plastics are used in the production of these crops in Europe (Scarascia-Mugnozza et al. 2011) and thus, this was considered to be a potential source of plastic to the catchment. Microplastic inputs from agricultural areas may also occur from the application of sewage sludge to land (Hurley and Nizzetto 2018). To assess the potential releases from this pathway, approvals for fertilising with sludge were obtained from the municipalities within the catchments of the five different lakes for the years 2017, 2018, and 2019. Data on the volume and date for sludge fertilising was extracted (Supplementary Material Table SI2). This was used as a proxy for potential microplastic inputs to the catchments.

### Data treatment and statistical analysis

All statistical analyses were carried out in the software R studio (version 4.1.0). Data were tested for normal distribution of residuals, by inspecting histograms and boxplots and testing with Shapiro–Wilk due to the small sampling size. A linear model was made to test if the concentrations of microplastic in the sediments (*n* = 5) were related to the environmental parameters. All environmental variables were tested for a relation to microplastic concentrations in sediment. Due to limited degrees of freedom, we could not use a linear mixed effect model with the lake as a random variable. All environmental parameters were tested two and two together with their interaction for combined effects. The models were tested for significance using an ANOVA test (*n* = 5).

A principal component analysis (PCA) combined with a biplot (method = Hellinger, scaling = symmetric) followed by the function envfit (Vegan package) was carried out to see if the polymer type of microplastic at the five sites could be explained by any of the environmental parameters.

## Results

### Sediment

A total of 42 particles were analysed in the blank samples. Of these, 37 particles were blue polypropylene fragments that were found to originate from the Falcon tube lid. All blue fragments matching those found in the blanks were removed from the data, as this was clearly contamination (see Supplementary Material, Figure SI7). The remaining five particles found in the blanks were viscose (1) and cellulose (4) fibres. As cellulose is not classified as microplastic and no viscose was observed in the environmental samples in this study, no action was taken to account for any additional procedural contamination. The limit of detection/limit of quantification (LOD/LOQ) was not calculated, based on the lack of microplastic in the blanks following exclusion of the blue polypropylene fragments. The recovery efficiency of the spiked material was 0.9 for beads and 0.7 for fragments.

Following FTIR analysis and contamination correction, a total of 31 particles (e.g. suspected paint, PP, PA, PC) detected in the sediment samples were confirmed to be microplastic (Table 2; for pictures of microplastic particles, see Supplementary Material Table SI3). This corresponds to generally very low concentrations of microplastics across all five lakes, with an average of 0.028 ± 0.017 items/g d.w. (Table 2). The highest concentration was found in Ravn sø (0.053 items/g), whereas the lowest concentration was found in Bryrup Langsø (0.009 items/g). Fragments were the most dominant particle type (77%; *n* = 24), and the remaining 23% were fibres. The particles’ longest dimensions varied between 99.2 µm and 2640 µm. Silkeborg Langsø and Hinge sø contained the largest microplastic particles, whereas sediment in Ravn sø, Arresø, and Bryrup Langsø generally contained smaller particles (see Supplementary Material Figure SI8).

**Table 2:** Results from the visual and FTIR analysis of sediment collected from five Danish lakes. For each lake are the concentration of microplastic in the sediment provided (items/g), total number of fibres in the sample, total number of fragments in the sample, and the identified polymer types

Lake	Items/g dry sediment	Total number of fibres	Total number of fragments	Number of different polymer types
Arresø	0.044	1	9	1 PS, 1 suspected paint, 7 alkyd varnish, 1 other
Bryrup Langsø	0.009	0	2	2 Alkyd varnish
Hinge sø	0.013	2	0	1 PA, 1 polyester
Ravn sø	0.053	1	11	9 suspected paint, 2 alkyd varnish, 1 polyester
Silkeborg Langsø	0.022	3	3	1 suspected paint, 1 PC, 1 polyester, 2 PP, 1 other

In Arresø, 78% of the microplastics were identified as alkyd varnish which is likely to originate from the boat activity in the lake (Wander et al. 2020). A number of particles found in Ravn sø visually resembled paint fragments and were subsequently recorded as ‘suspected paint’ based on the outcome of the FTIR analysis, which cannot typically unanimously verify polymeric paint particles (Käppler et al. 2016).

The PCA was carried out to test if the variance in polymer types could be explained by the environmental variables. The PCA followed by an envfit analysis showed that the environmental variables do not significantly explain the variance in polymer types found in the sediment in the five different lakes (*p* = 0.05, *n* = 5). Potential plastic cover in the catchment showed to be a slightly significant explanatory variable (*p* = 0.042) (see Supplementary Material Figure SI9, Table SI4, and Table SI5); however, the polymer types detected in the sediment do not match the common polymer (PE) used in agricultural production in Europe.

### Mussels

Despite a total of 150 mussels being analysed for microplastic content (30 mussels from each of the five lakes), only a single microplastic particle was detected following visual analysis and chemical characterisation. No microplastic was detected in the blank samples processed with the mussels, indicating no procedural contamination and preventing the opportunity to establish LODs or LOQs for this data. The recovery efficiency of the spiked material was 1 (± 0 standard deviation (S.D.)) for fibres, 0.92 (± 0.098 S.D.) for fragments, and 0.96 (± 0.049 S.D.) for beads, indicating sufficiently high recovery of a range of particle types.

The single microplastic was detected in a composite sample replicate (10 individuals) from Silkeborg Langsø. It was a blue polypropylene fragment, 91.8 × 69.3 µm in size. The particle did not visually resemble the particles identified in the sediment samples that originated from the Falcon tube lids SI Fig. 14). Falcon tubes were not used in the processing of the mussel samples and the samples were processed separately. The single particle corresponds to a concentration of 0.067 items/10 individual or 0.02 items/gram (w.w.), although it is recommended to treat these data with caution based on the single data point. No microplastics were detected in any other mussel samples from all other lakes in this study, indicating negligible microplastic occurrence in *D. polymorpha* in Danish lakes for particles > 50 µm.

### Relationship between environmental data and microplastic concentrations

Full details of the environmental parameters (i.e. annual discharge from effluent point sources, agricultural practices, and in-lake biochemical data) used in the analysis can be found in Supplementary Material 6, Figures SI11, SI12, SI13, and SI14, and Tables SI6, SI7, SI8, and SI9.

There was no significant relationship between microplastic concentrations in the sediments and any of the following environmental variables (Lm model *p* > 0.05): TN and TP, prevalence of crops potentially subject to agricultural plastic use, volume of sludge used in the catchment as fertiliser, discharge from effluent point sources measured as COD. Potential correlations between sediment microplastic concentrations and multiple environmental parameters and their interaction were also tested, but no significant relationships were found (lm model *p* < 0.05). In addition, no correlations could be found when testing based on individual polymer types (*p* < 0.05). A PCA analysis was carried out and the contribution of each factor was assessed using the function envfit (vegan package, permutations = 1000, scaling = ‘sites’, choices = 1:2). Area in catchment with prevalence of crops potentially subject to agricultural plastic use presented a slight significant relationship (*p* = 0.042).

## Discussion

### Microplastic pollution in lake sediments

Based on the environmental context of the lakes included in this study, in addition to the evidence for widespread microplastic contamination even in remote lakes with few potential microplastic sources, microplastic contamination could be expected to occur in these locations. All five lakes and their catchments possess several potential microplastic sources including agricultural activity, input from effluent point sources, and proximity to urban areas. The five Danish lakes were found to have low sediment concentrations compared to the microplastic concentrations published by the majority of other studies on a global scale (median value of 385 items per kg) (Dusaucy et al. 2021). Similarly, comparing the concentrations of microplastic found in the five Danish lakes with a comprehensive study of microplastic in the sediments of the Norwegian lake Mjøsa (without a high anthropogenic influence; average 0.067 items/g d.w.) Clayer et al. (2021) showed that the concentrations in the Danish lakes (average (0.028 items/g d.w.) are lower than the concentrations found at the natural sites in Lake Mjøsa. Townsend et al. (2019) carried out a study in 20 urban wetland sediments around and within Melbourne, Australia, and found that wetlands with a catchment dominated by open areas had the lowest microplastic concentrations compared to catchments that were more heavily urbanised, which has also been reported by Yonkos et al. (2014) for rivers and estuaries spanning a gradient in population densities within the Chesapeake Bay, USA. These results thus support the low concentrations in the present study, where catchments are dominated by open areas.

The three samples from each lake are thought to represent the sampling area well; however, different geographical and hydrological conditions exist in the lakes, and including more samples from other sites within the lakes would have been better to represent the microplastic contamination of the entire lakes. No relationship between the sediment microplastic concentrations and any of the environmental parameters or their interactions was found. We originally hypothesised that there would be a relationship between potential catchment sources of microplastics and sediment concentrations. The rejection of this hypothesis indicates the complexity associated with sources of microplastic contamination. On the other hand, the lack of a relationship might also be a result of the very low concentrations, indicating that no major source exists, and thus making the contribution from different sources difficult to quantify. The analysis could benefit from additional data points: having more degrees of freedom would allow us to test for more correlations and potential interactions. Understanding of the way in which microplastic particles distribute spatially in lake sediments is still in its infancy. Thus, it is possible that the sampling locations in this study represent cold spots with minimal deposition of microplastic, whereas higher concentrations could possibly exist in other parts of the lakes. Nevertheless, taking composite samples in five different lakes reduces the likelihood that cold spots were sampled in all locations. Further investigation into the mechanisms that govern microplastic distributions in the environment is essential to effectively interpret observed microplastic concentrations.

Most Danish lakes are impacted by eutrophication caused by excess loading of nutrients (Jeppesen et al. 2007) and only 25% fulfil their environmental objective of good ecological status (Miljøstyrelsen 2020). Our study suggests that resources should be targeted to reduce nutrient loading rather than focus on quantifying the presence and potential impacts of microplastics if the goal is to improve ecological conditions in Danish lakes. It appears that sources and subsequent accumulation of microplastic are not closely related to drivers of eutrophication suggesting that these environmental issues should be tackled separately.

### Potential microplastic sources

It is notoriously difficult to link observed microplastic assemblages to specific sources, especially considering their diversity and the environmental complexity connecting potential sources to the specific sampling location. Yet, this study identified suspected paint particles which could originate from recreational activities such as sailing, canoeing, and kayaking. Recreational activities are difficult to quantify and can be localised. Thus, it is important in future studies to sample the shores around the entire lake to get a representative sample (Dean et al. 2018). Beyond this, it was not possible to attribute other particles to other potential specified sources.

Instead, it is notable that the lake catchments are characterised by a large number of potential microplastic sources whilst concentrations in lake sediments were low at the site sampled in each lake. Higher concentrations elsewhere in the lake could be possible, related to the scale of potential spatial variability in lake systems (Stanton et al. 2020). Bryrup Langsø has, on average over the last 3 years, received the largest amount of sludge in the catchment; yet, this lake has the lowest microplastic concentration in the sediment. In contrast, Ravn sø has the second highest concentration of microplastics in the sediment and yet has a catchment where sludge has been deployed in only relatively low quantities. Based on the inability to specifically verify the use of agricultural plastics in the catchments, it is difficult to assess the potential input of microplastic from this source, even though the particle types identified do not match the common forms of agricultural plastic used in Europe. Our findings suggest that reporting proxies for agricultural activities—which may be associated with microplastic release—is not sufficient to explain sediment microplastic concentrations downstream in the catchment. Agricultural practices, including sludge application to land and use of agricultural plastics, could represent a potentially large source of microplastic to soil environments, and research quantifying this for different geographic regions is still underway. Specific assessment of potential sources of microplastic in these catchments is required to fully assess the use of different proxies. Furthermore, studies which utilise similar proxies for agricultural activities should consider additional complexities, such as the connectivity of agricultural land to freshwater systems and the extent of microplastic erosion from farmed soils. Some studies have identified microplastic particles derived from agricultural activities in freshwater environments, such as in the Ofanto river, Italy (Campanale et al. 2020). Yet, recent research has also highlighted extremely high variability in the net export of microplastic from agricultural soils, which can be dependent on soil type, local environment, and specific agricultural practices. For example, Crossman et al. (2020) report that 99% of microplastics applied via sewage sludge are lost from fields in Canada over the course of a year, whilst 99% of microplastics were retained in the soil in Spain after 1 year (Schell et al. 2021). Thus, microplastic input from agricultural sources may accumulate in soils and there may not be widespread transfer of particles to aquatic systems. This is likely to be dependent on a suite of soil and catchment characteristics, which have not yet been elucidated in the literature. Further research is needed to better understand the fate of agriculture-derived microplastics in the wider environment.

Discharge of sewage from point sources represents an additional potential source of microplastics to the lake catchments. It is possible that the higher concentrations observed in Ravn Sø could be explained by the higher density of outlets per lake area. Combined with a high retention time of this lake (Table 1) resulting in low flow velocities and thus higher sedimentation rates (Dahms et al. 2020), this could encourage microplastics to accumulate in the lake sediment. However, sedimentation processes affecting microplastic are likely highly complex—depending on density, hydrology, morphology, etc.—and are not yet completely understood (Daily and Hoffman 2020; Vaughan et al. 2017). Our statistical analyses did not reveal a significant correlation between COD and microplastic concentration or COD and its interaction with retention time and microplastic concentration. We also found a higher prevalence of fragments over fibres, which is contrary to several studies documenting predominant fibre release from wastewater treatment systems (Clayer et al. 2021, Mahon et al. 2017).

Yet, as with potential agricultural sources, it is important to recognise that using proxy data for potential sources may not be straightforward. For example, the ten largest wastewater treatment plants in Denmark, managing 26% of Danish waste water, are extremely efficient in retaining microplastics, with 99.3% of particles removed from the water (Simon et al. 2018). This suggests that wastewater treatment may not constitute a substantial source of microplastics to the Danish freshwater environment. Simon et al. (2018) relate the COD/L discharged by WWTP to microplastic per L (MP/L) and find that the microplastic mass concentration is 7.80·10^−4^ to 3.37·10^−4^ mg MP/mg COD. Assuming that the studied lakes receive discharge from WWTP of the same quality as the ten WWTP analysed by Simon et al. (2018), we can apply the relationship between COD and microplastic concentration to the data of COD from effluent point sources to the studied lakes. Performing this calculation results in an annual microplastic load of between 0.16 and 176.23 kg MP/km^2^/year (Table 3). If calculated per sampled area, it would correspond to a maximum deposit of 0.01 µg per year at Hinge sø and 3.42 µg per year in Silkeborg Langsø (Table 3). These values are very low, supporting the low concentrations we observe in the analysed sediment.

**Table 3:** Calculated discharge on microplastics (MP) based on COD to the five Danish Lakes and the relationship between COD and MP given in Simon et al. (2018)

Lake	Calculated MP load (kg/km^2^/year) based on COD load to the catchment and Simon et al. (2018)	Calculated MP load (kg /km^2^/year) based on COD load directly to the lake and Simon et al. (2018)	MP values deposited per sampling area of sediment per year (µg/year)	Max values let directly into the water (µg/m^3^/day)	Max values in water (corrected for retention time (µg/m^3^)	Estimated ingested particles per day per mussel (if no sedimentation happened, and if all particles were available for 1 single mussel)
Arresø	2.98–6.98	0.20–0.46	0.04	0.41	460.69	0.02–0.48
Bryrup Langsø	6.45–15.10	0.16–36.9	0.08	6.74	492.24	0.02–0.51
Hinge sø	0.85–1.00	1.57–3.68	0.01	25.74	469.82	0.02–0.49
Ravn sø	10.94–25.62	1.85–4.32	0.15	0.08	70.82	0.003–0.07
Silkeborg Langsø	248.07–580.79	75.27–176.23	3.42	78.90	935.92	0.04–0.97

Proxy data to indicate potential sources of microplastic have been used to explain microplastic concentrations in many studies. The findings presented here show that often the situation is complex, and more specific quantifications of source contributions are required to identify all sources of microplastic to freshwater systems and apportion concentrations. Yet, it is also important to note that proximity to sources is not the only factor that determines observed microplastic distributions in the environment. Environmental processes that govern the fate and transport of particles are also relevant to explain levels observed in different environmental matrices and across different spatial and temporal scales. There may be mechanisms which limit widespread release, encourage flushing of particles, or sustain particles in suspension, preventing sedimentation (Horton et al. 2017b; Hurley et al. 2018a; Lambert and Wagner 2018). Research is required to shed a light on fate and transport processes for a range of environmental scenarios (Yang et al. 2020).

### Absence of microplastic in Dreissena polymorpha

Li et al. (2018) listed a set of criteria for a suitable monitoring species which included the capability to filter high volumes, having a wide geographical occurrence, dominantly sessile, high population densities, and a well-known biology. Thus, the ecology of freshwater bivalves indicates that they may have potential to act as a bioindicator for microplastic pollution in the surrounding environment. The use of bivalves as an indicator species in particular in freshwater systems requires an assessment of the individuals themselves but also a comparison to abiotic matrixes. A previous study of *D. polymorpha* confirmed that microplastic was ingested in the natural environment and the authors suggested that it could potentially act as a sentinel for microplastic contamination (Pastorino et al. 2021). They reported relatively low concentrations of between 0.03 and 0.23 item/individual (organisms were of a corresponding size range as in this study). In order to test the suitability of sentinels or bioindicators, they must be compared to other abiotic and biotic samples collected in the same area.

Only a few studies have published negative results for microplastic presence in relation to freshwater organisms. This may be in response to a publication bias towards positive results, despite the fact that negative results are often also valuable contributions to improve our understanding of the ecological impact that microplastic may have (Toner and Midway 2021). To our knowledge, only two studies worldwide have thus far reported a lack of microplastics in freshwater benthic invertebrates (chironomids and two other *Dreissena* species) (Pastorino et al. 2020; Schessl et al. 2019). The lack of microplastic in chironomids found by Pastorino et al. (2020) was in agreement with the general absence of microplastic in the high alpine lake in which they were sampled. Schessl et al. (2019) studied 149 dreissenid mussels for microbeads, but none was observed, even at sites, where beads were found to be numerous in the surrounding sediment. The authors suggested that the absence of beads in the mussels was due to the size of the microplastic, limiting ingestion.

Organism size may play a significant role when considering why microplastics were not detected in *D. polymorpha*. The relationship between bivalve size and microplastic ingestion is not clear, and several studies have failed to find any significant relationship between body size (i.e. length or weight) and ingestion rate (Digka et al. 2018; Scherer et al. 2017; Wardlaw and Prosser 2020). Yet, Phuong et al. (2018) found that larger bivalves contained more microplastic than smaller ones, whilst Doucet et al. (2021) reported a negative correlation between body size of the freshwater mussel *Margaritifera margaritifera* L. and the number of ingested microfibers. Hoellein et al. (2021) found that *Dreissena polymorpha* < 1 cm had significantly less microplastic per individual than organisms with a size between 1 and 2.5 cm, and when reported per gram wet weight, the size class with the highest concentration was the size class 1–1.5 mm whereas mussels < 1 cm and > 2.5 had the lowest concentration. This supports the chosen size of mussels for this study, as well as the fact that they are more numerous than larger individuals and thus are easier to sample—and they have a higher filtration rate. For example, Reeders and De Vaate (1990) showed that the filtration rate of *D. polymorpha* decreases for individuals above 22 mm. More research is required to understand the thresholds and relationships related to organism size and microplastic ingestion to better inform about the potential for bivalve species to represent suitable bioindicators in freshwater systems.

Another potential reason for the absence of microplastic in the mussels could be that they preferentially ingest smaller sized microplastic, and that these particles fall below the analytical detection limit in this study (50 µm). The size threshold at which *D. polymorpha* no longer ingests microplastic or expels them through pseudofaeces is not yet known. Researchers have presented conflicting information surrounding this topic. Naddafi et al. (2007) suggested that everything above 50 µm is expelled; however, larger particles have been observed in *D. polymorpha* sampled in the field (Pastorino et al. 2021). Magni et al. (2018) found that polystyrene beads of 1 µm and 10 µm were ingested but did not test larger sizes. In another study, *Dreissena* were exposed to microplastic in the size fraction < 63 µm, where 90% of the particles were < 12.4 µm. This study too showed that microplastics are ingested by *Dreissena* under laboratory conditions, but provided no additional information about the size of the particle which were ingested (Weber et al. 2020). Evidence from field studies observed particles 149 µm to 2289 µm in *D. polymorpha* (Pastorino et al. (2021). The capacity to ingest particles in the range 900–1200 µm was further confirmed by Horgan and Mills (1997). The smallest particles detected in the sediment in this study (based on longest dimension) were 90 µm and should therefore be theoretically available for ingestion by *D. polymorpha*. A list of in situ studies on freshwater bivalves and the size of ingested microplastic particles can be found in Supplementary Material Table SI10. It is possible that with a lower size detection limit, higher concentrations may have been detected in the sediments and, potentially, the mussels. Nevertheless, current microplastic monitoring focuses on larger microplastics particles, typically in the range > 300 µm, so species that preferentially ingest particles below the detection limit here (50 µm) may not represent a suitable bioindicator species in this context (Lusher et al. 2021b). Finally, the lack of microplastics detected in *D. polymorpha* in this study may reflect the low concentrations observed. This represents limited exposure to microplastics in these settings. The extent to which microplastics may have been initially ingested and expelled in pseudofaeces is also unknown. No study has, to our knowledge, shown the extent and composition (size, shape, polymer) of microplastics that are egested as pseudofaeces.

The average volume of the particles for all five lakes was 0.0050 (± 0.042 S.D., min = 0.00068, max = 0.22) cm^3^. Calculating with an average density of 1, this corresponds to an average weight per particle of 5.02 µg. Comparing those values to the calculated discharge directly into the lake from effluent point sources based on Simon et al. (2018) corresponds to a release of 0.08–78.90 particles per m^3^ per day. *D. polymorpha* has a clearance rate between 0.018–0.432 L/mussel/h (Baldwin et al. 2002), corresponding to between 0.00043 and 0.009648 m^3^/mussel/day. Thus, the estimated ingestion of particles per mussel per day is between 0.003 and 0.97 particles. This, in combination with the fact that some particles will sediment out, that multiple individuals filter the same volumes of water, and that *D. polymorpha* can produce pseudofaeces containing particles which are not suitable as food items, supports the results of this study. Very few particles will be available for consumption for a single mussel and when ingested, it might be expelled.

From the finding presented in this current study, it is not possible to confirm that *D. polymorpha* interact with microplastic in Danish lake ecosystems. Neither is it possible to discern that microplastics accumulate in the mussels under low exposure conditions. The lack of microplastics detected in the mussels and the low environmental concentrations does not preclude the suitability of *D. polymorpha* as a potential bioindicator; however, more research is required to provide a more detailed assessment of the thresholds governing interaction between microplastics and freshwater bivalves.

## Conclusion

Despite an array of potential microplastic sources, five lakes in impacted catchments in Denmark revealed very low sediment concentrations (average 0.028 ± 0.017 items/g d.w. sediment (*n* = 5)). Contamination from the Falcon tube lid was observed in the blanks and samples, and these were not included in the final concentrations. Only a single particle was detected in the freshwater mussel *D. polymorpha* across all five lakes (average 0.067 ± 0.249 items/10 individual, *n* = 150). No relationships were found between sediment microplastic concentrations and a series of environmental parameters nor between environmental data (e.g. TN, TP, COD discharge to lake and catchment, application of sludge, potential plastic cover) and polymer types in the sediment, except for plastic cover, which was in turn not supported by the qualitative data of microplastic characteristics. This sheds a light on the difficulties associated with identifying and apportioning microplastic sources in catchments, where many of the actual inputs are expected to be spatially and temporally dynamic, and may be diffuse in nature. Many sources have the potential to deliver microplastic to freshwater systems (e.g. industry, wastewater effluent, agriculture, urban areas, roads) but more research is needed to elucidate and quantify the release pathways over a range of spatial and temporal scales. This study also demonstrates that using proxy data (COD discharge, plastic cover, sludge application, and water chemistry) for potential sources (agriculture and urban areas) may not sufficiently explain observed environmental concentrations and emphasises that future studies should quantify actual sources of microplastic in the catchment. The study thus contributes to knowledge on interactions between catchment, lake, and biota at low microplastic concentrations. In addition, the study contributed to ongoing debates regarding optimal sampling methods, e.g. number of locations, sample size, and size of mussels when analysing for microplastics. More research is required to better understand why microplastic concentrations in these impacted lakes are low compared to other studies in similar and comparatively pristine catchments.

## Supplementary Information

Below is the link to the electronic supplementary material.

Supplementary file1 (DOCX 18662 KB)

## Data Availability

The datasets used and/or analysed during the current study are available from the corresponding author on reasonable request.
