# Supplementary material for: Anthropogenically impacted lake catchments in Denmark reveal low microplastic pollution
Source: Environ Sci Pollut Res Int. 2022 Feb 19;29(31):47726–39. doi: 10.1007/s11356-022-19001-8 (PMC9232414; doi:10.1007/s11356-022-19001-8)
Supplement: Supplementary file 1 — Supplementary file1 (DOCX 18662 KB) [file 11356_2022_19001_MOESM1_ESM.docx]

Supplementary material

## Title: Highly agricultural and populated lake Anthropogenically impacted lake catchments in Denmark reveal low microplastic pollution

## Journal name: Environmental Science and Pollution Research

## Author names: Emilie M. F. Kallenbach1,2, Nikolai Friberg1,2,3,4, Amy Lusher3,5, Dean Jacobsen2 & Rachel R. Hurley3

## Affiliation:

## 1) NIVA Denmark Water Research, Njalsgade 76, 2300 Copenhagen S, Denmark

## 2) University of Copenhagen, Universitetsparken 4, Copenhagen Ø, Denmark

## 3) NIVA, Økernveien 94, 0579 Oslo, Norway

## 4) University of Leeds, water@leeds, School of Geography, Leeds LS2 9JT UK

## 5) Department of Biological Sciences, University of Bergen, 5020 Bergen, Norway

## E-mail address of the corresponding author: eka@niva-dk.dk

# Maps and pictures of sampling sites

The sediment and mussel samples were collected in five lakes in Denmark (Figure 1) For each site a orthophoto of the lake and a picture of the sampling site from the sampling date can be found (Figure 2-6)


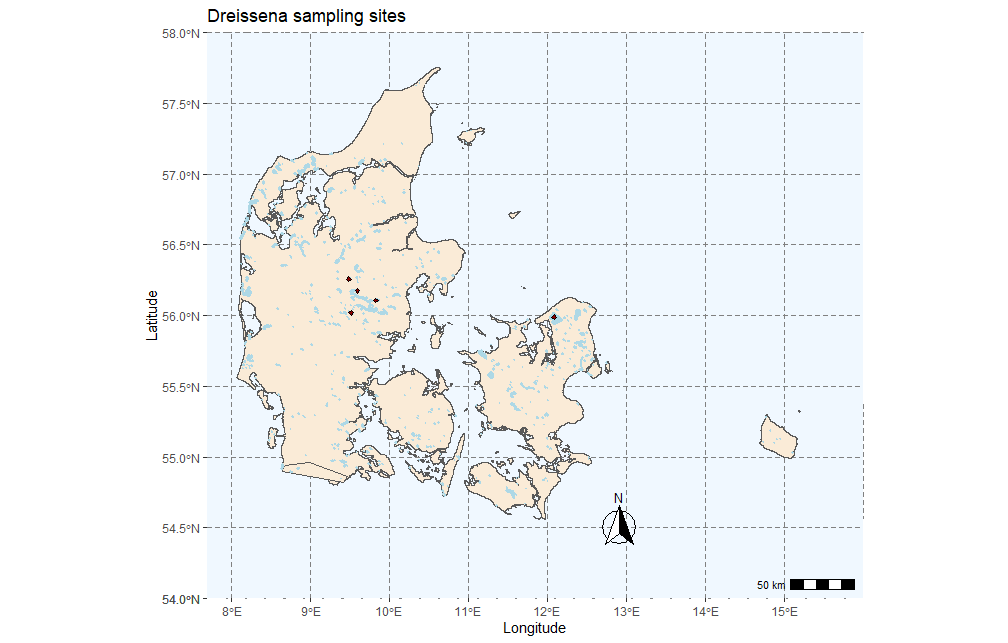

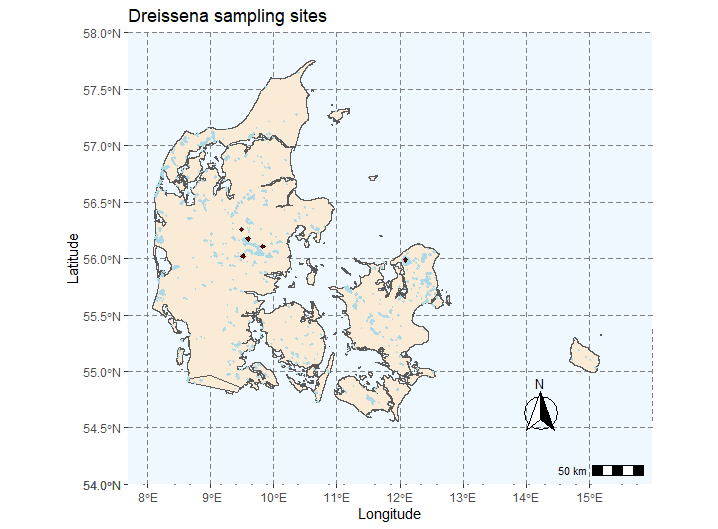


*Figure 1. Map of Denmark showing the five sampling sites.*


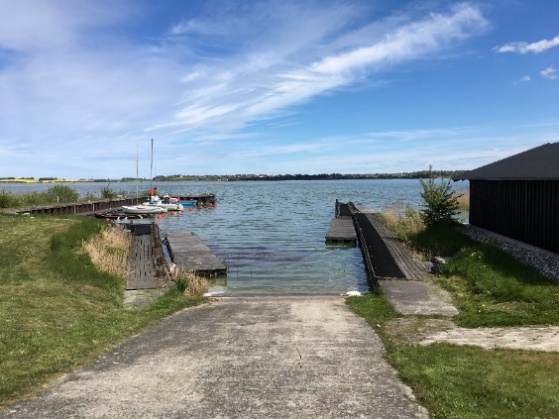

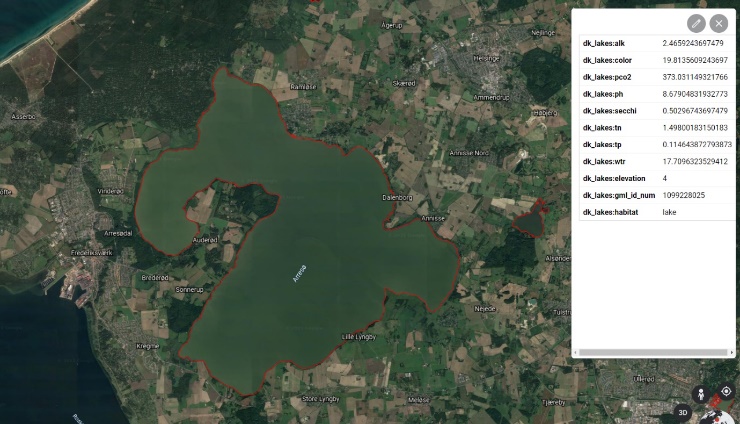


*Figure SI2: Sampling site and orthophoto of Lake Arresø*


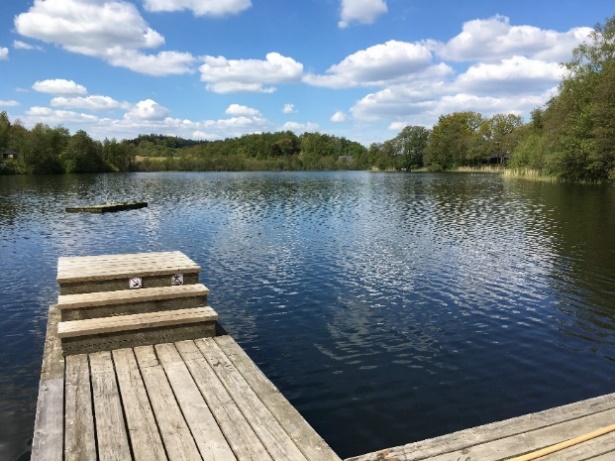

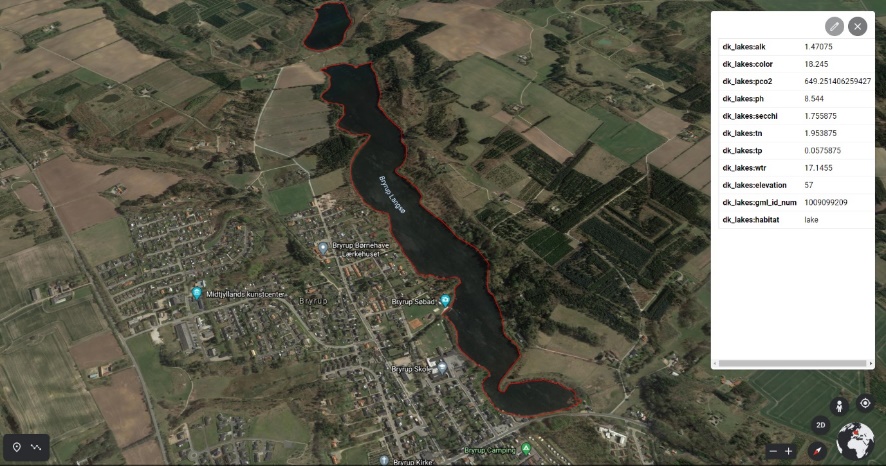


*Figure SI3: Sampling site and orthophoto of Bryrup Langsø*


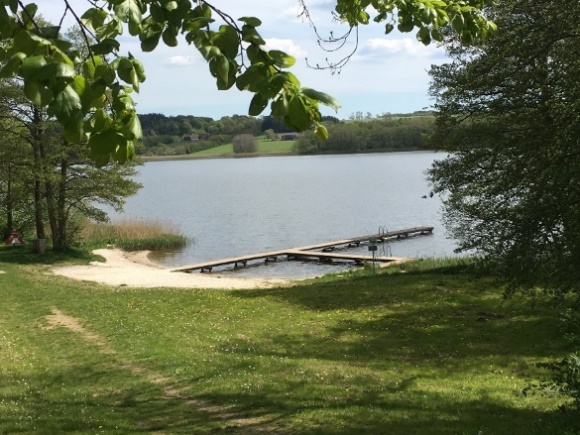

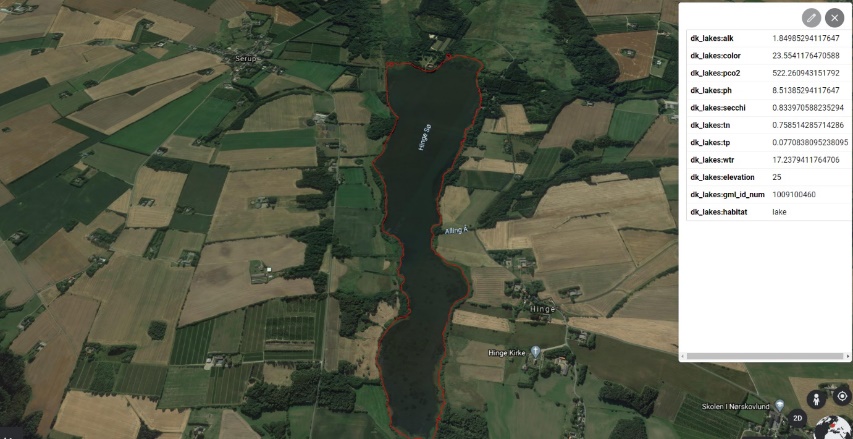


*Figure SI4: Sampling site and orthophoto of Hinge Sø*


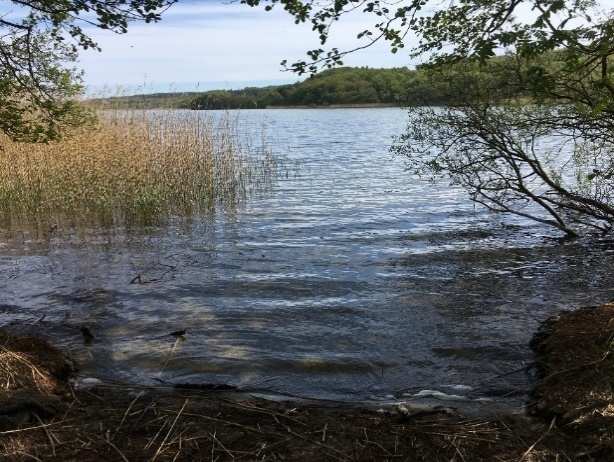

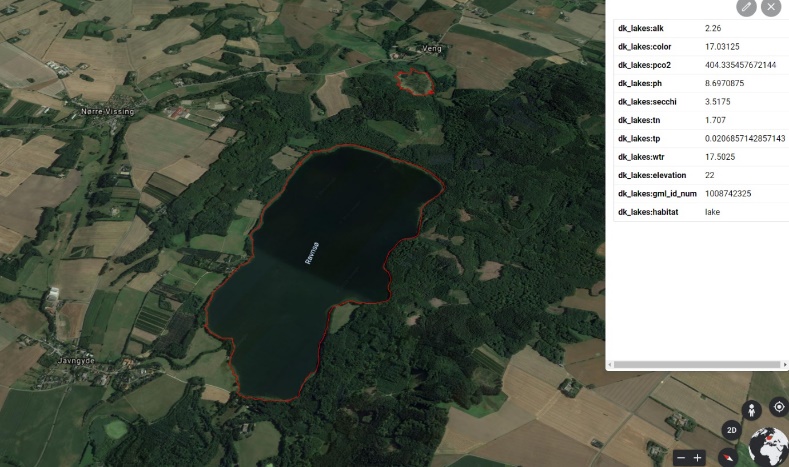


*Figure SI 5: Sampling site and orthophoto of Ravn Sø*


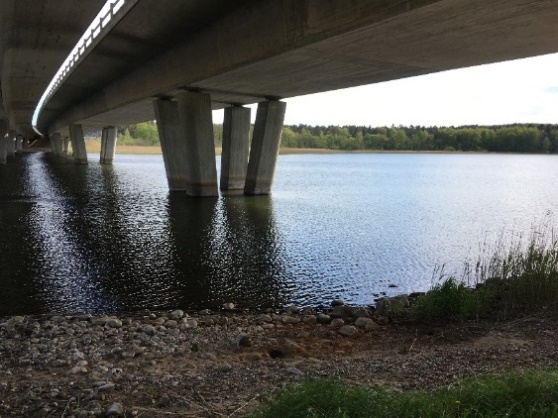

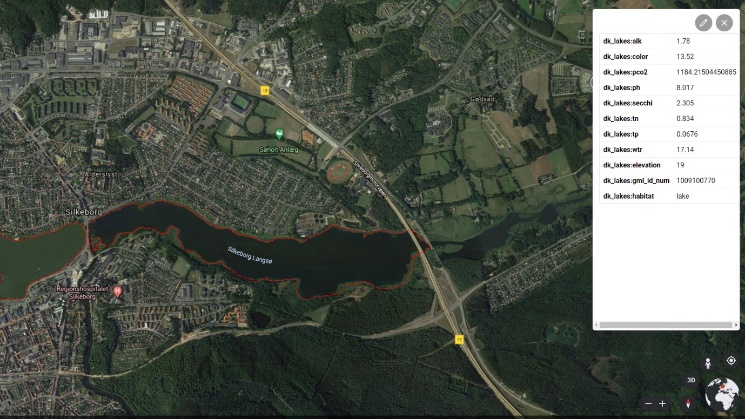


*Figure SI 6: Sampling site and orthophoto in Silkeborg Langsø*

# Data from sampling

Sampling took place two days in May 2019. At each site three sediment cores were sampled and 30 mussels. The coordinates for sampling location and size of the mussels are provided in Table 1

*Table SI1: Data from sampling of sediment and mussels at the different sampling sites.*

| *Lake* | *Coordinates* | *Sampling date* | *Sediment type* | *Substrate for mussels* | *Average wet weight mussels (g)* | *Average length mussels (cm)* | *Average width mussels (cm)* |
| --- | --- | --- | --- | --- | --- | --- | --- |
| Arresø | 55.988668, 12.083813 | 14.05.2019 | Soft bottom | Wooden pier | 0.2 | 1.8 | 0.8 |
| Bryrup langsø | 56.020415, 9.516921 | 15.05.2019 | Soft bottom | Wooden pier | 0.3 | 1.8 | 0.9 |
| Hinge sø | 56.257527, 9.488302 | 15.05.2019 | Soft bottom | Wooden pier | 0.4 | 1.9 | 1.0 |
| Ravn sø | 56.105926, 9.828211 | 15.05.2019 | Soft bottom | Large stones on bottom | 0.5 | 2.2 | 1.0 |
| Silkeborg langsø | 56.174934, 9.597014 | 15.05.2019 | Soft bottom | Stones at the sediment surface | 0.4 | 2.1 | 1.1 |

# Data from sampling

| **Lake** | **Mean** | **S.D.** |
| --- | --- | --- |
| Arresø | 98.8 | 44.14834 |
| Bryrup Langsø | 1315.8 | 3721.615 |
| Hinge sø | 26.9 | 76.03299 |
| Ravn | 461.8 | 191.755 |
| Silkeborg Langsø | 152.4 | 114.217 |

*Table SI2: Fertilising with sludge in the catchment of the 5 lakes. Mean and standard deviation of the three years 2017, 2018 and 2019 are given.*

# QAQC

Blue PP fragments from the falcon tube lid was found in the blank samples. Blue PP fragments similar to these, that was found in the sediment samples was not included in the results. Pictures of particles and chemical images from FTIR can be found in figure SI

|  | Picture of particle | FTIR spectrum of particle |
| --- | --- | --- |
| Blank | 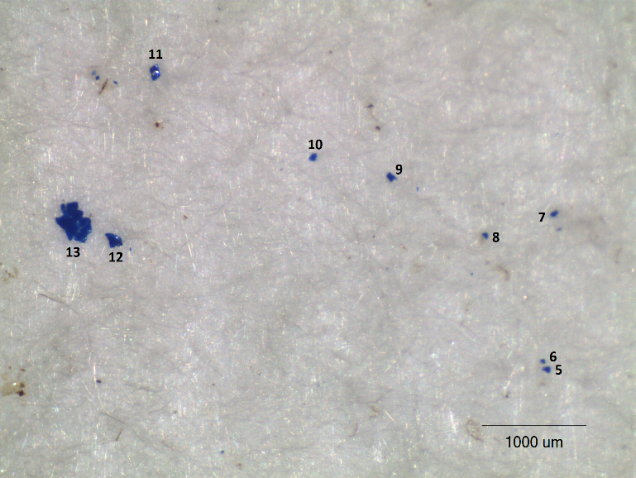 |  |
| Sample from Silkeborg Langsø | 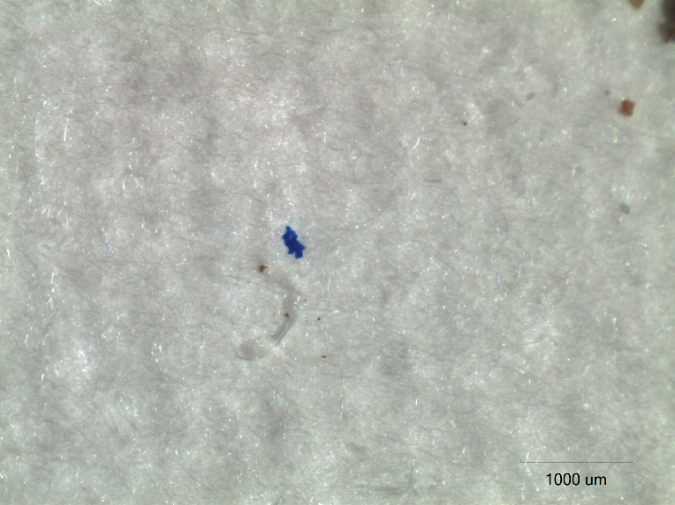 |  |

*Figure SI7: Blue polypropylene fragments from deriving from the Falcon tube lid from the blank sample and from a sediment sample and their chemical spectra.*

# Results:

# Pictures of particles

*Table SI3: Pictures of all microplastics found in the sediment. First column provide numbers of the particles that were identified as plastic polymers. Numbers not mentioned, were either contamination or not plastic polymers.*

| **Particles identified as MP** | **Arresø** |
| --- | --- |
| No 2 | 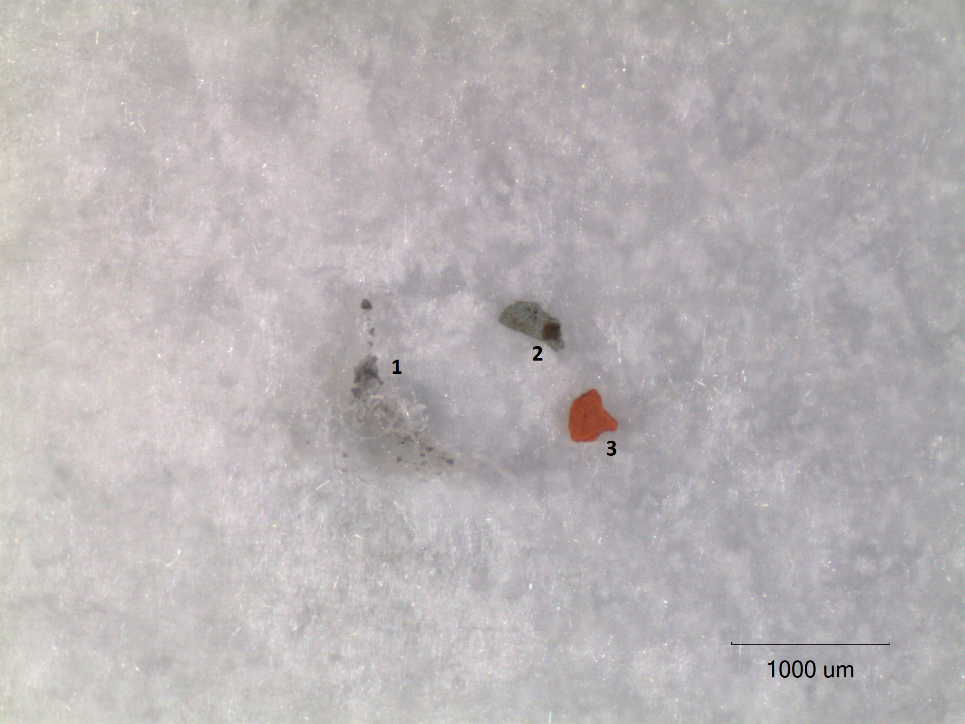 |
| No. 7, 8, 10 | 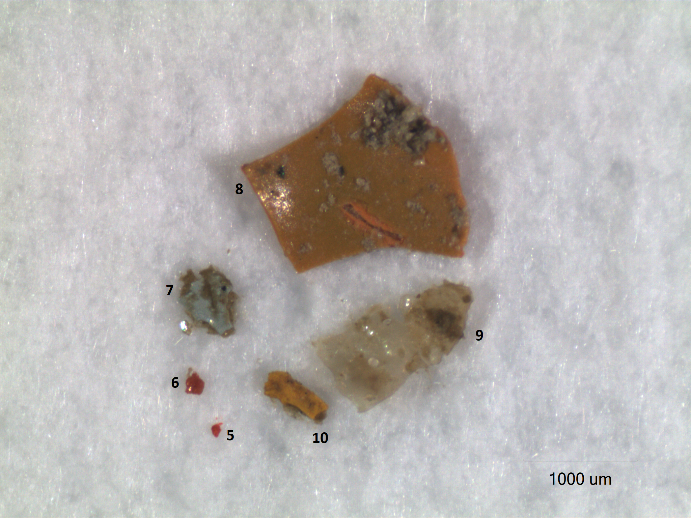 |
| No. 11, 12, 13 | 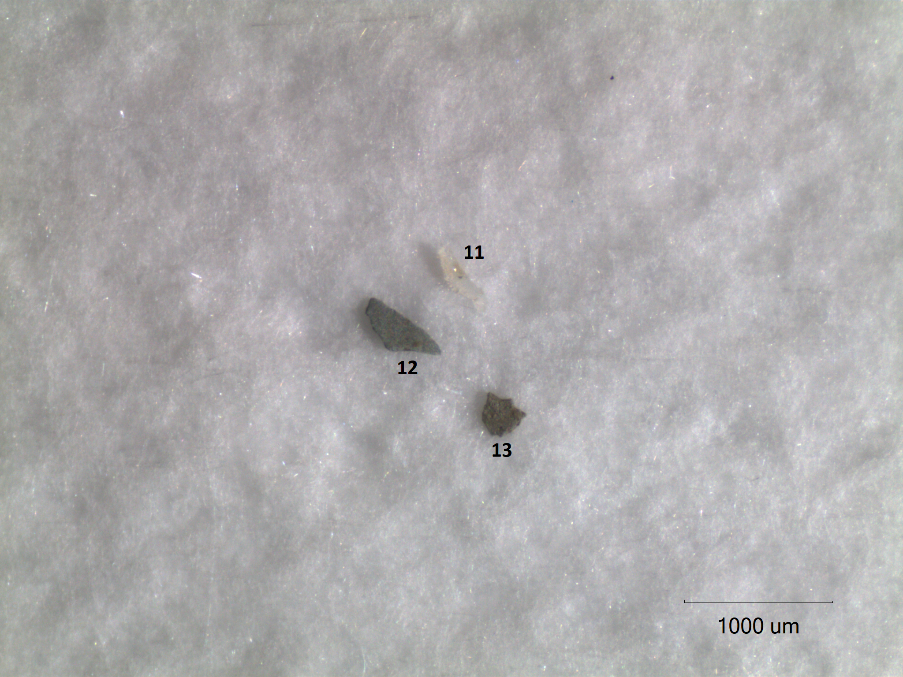 |
| No. 14, 15. | 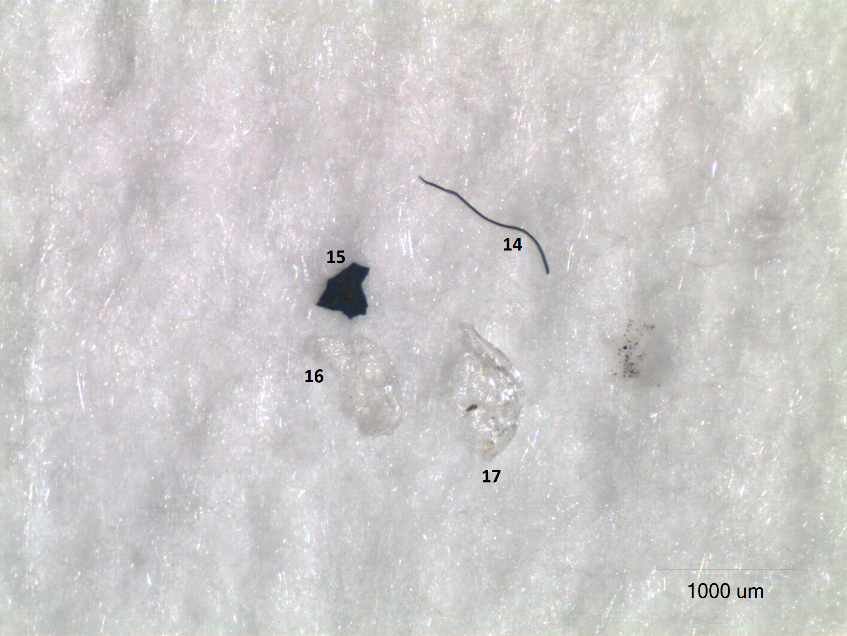 |

| **Particles identified as MP** | **Bryrup langsø** |
| --- | --- |
| No. 3, 4 | 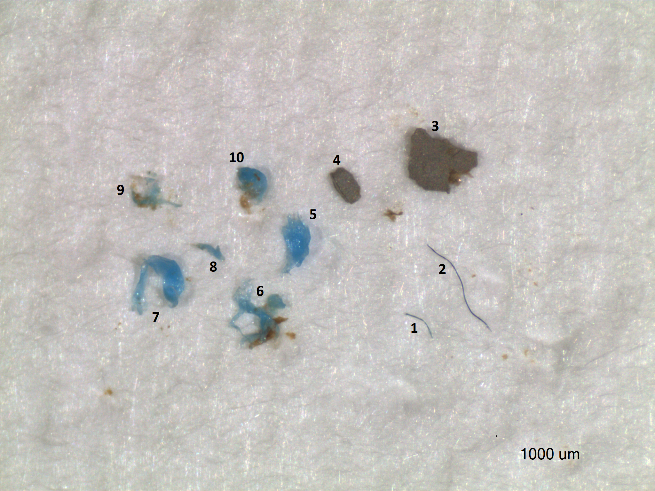 |

| **Particles identified as MP** | **Hinge Sø** |
| --- | --- |
| No. 1 | 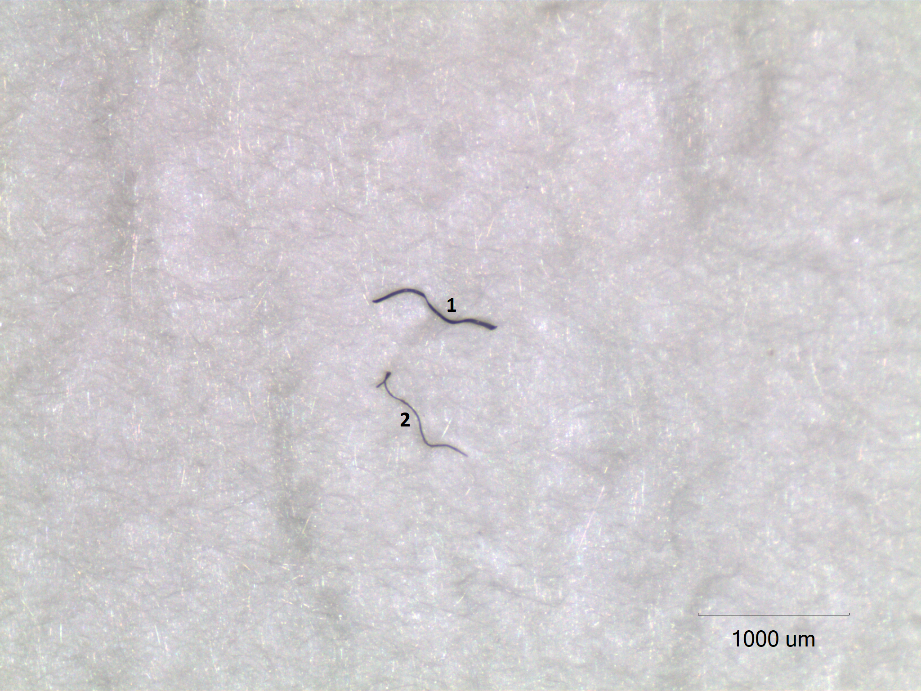 |
| No. 13 | 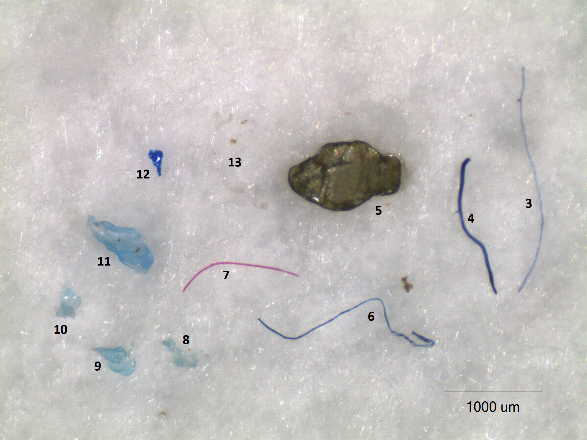 |

| **Particles identified as MP** | **Ravn Sø** |
| --- | --- |
| No. 4, 5, 7 | 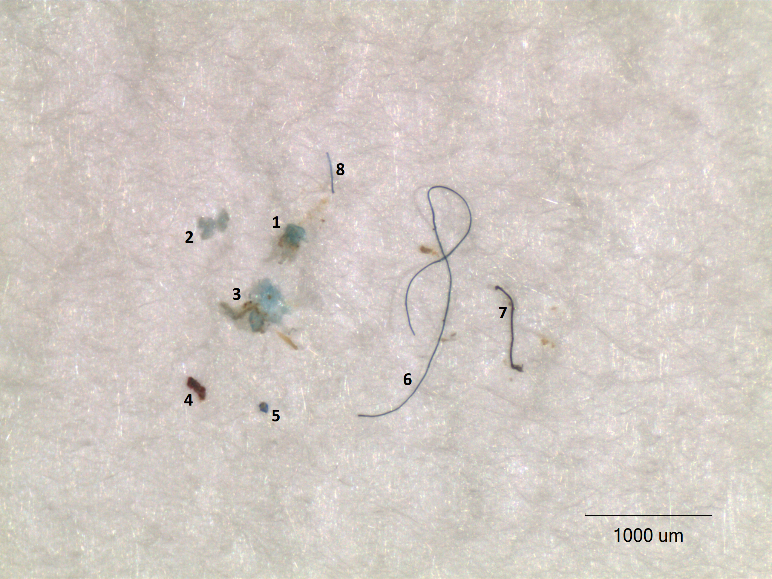 |
| No. 11, 12, 14, 15, 16, 17, 18, 19 | 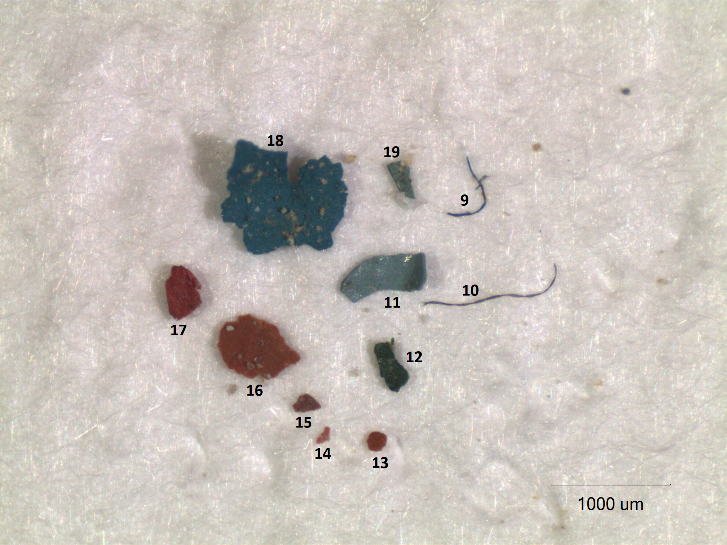 |
| No. 24 | 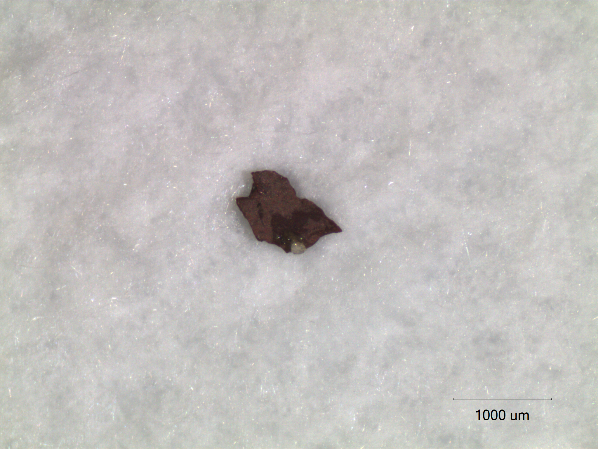 |

| **Particles identified as MP** | **Silkeborg Langsø** |
| --- | --- |
| No. 4 | 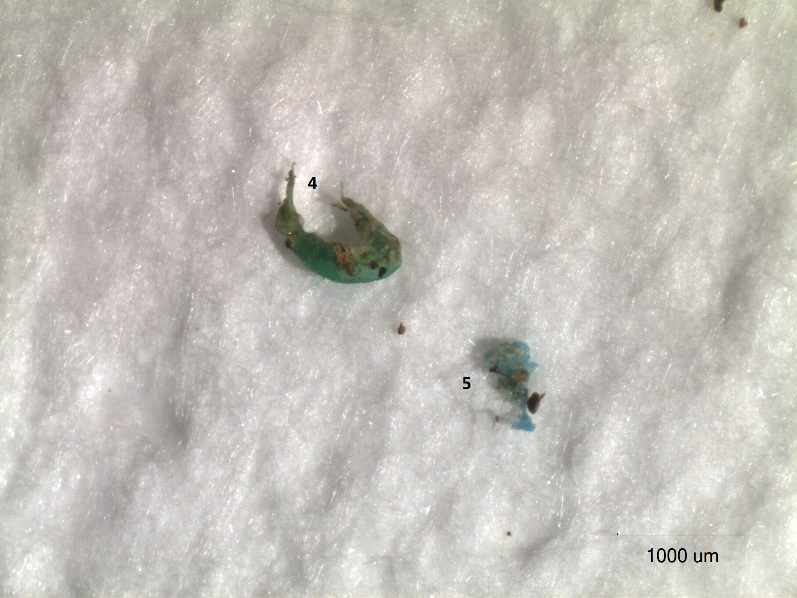 |
| No. 14, 16, 18 | 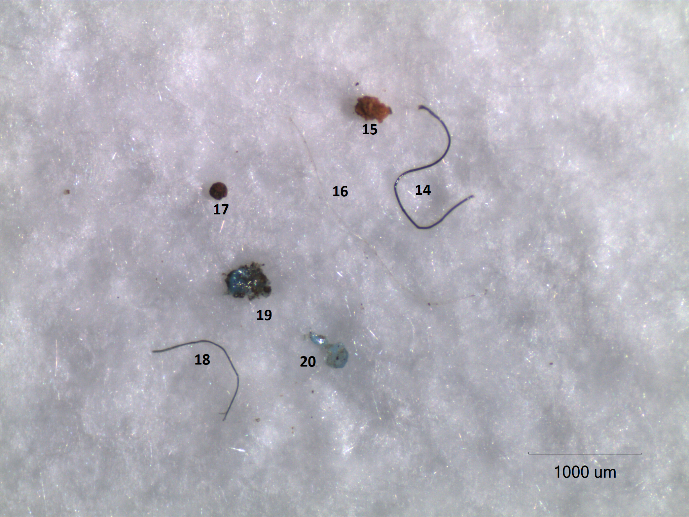 |
| No. 32 | 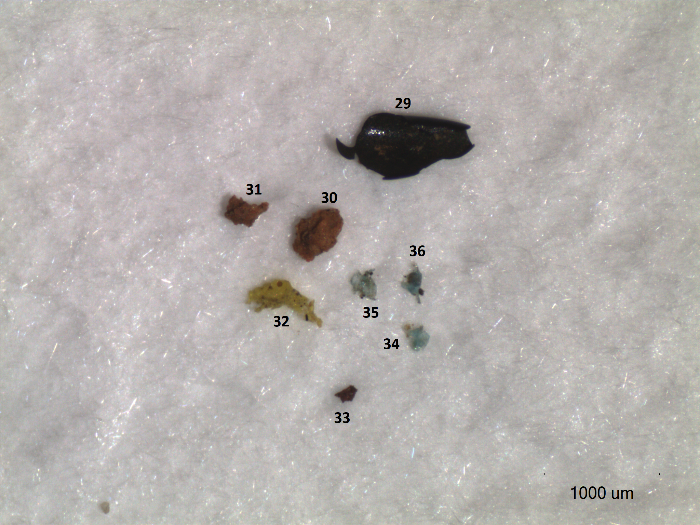 |
| No. 37 | 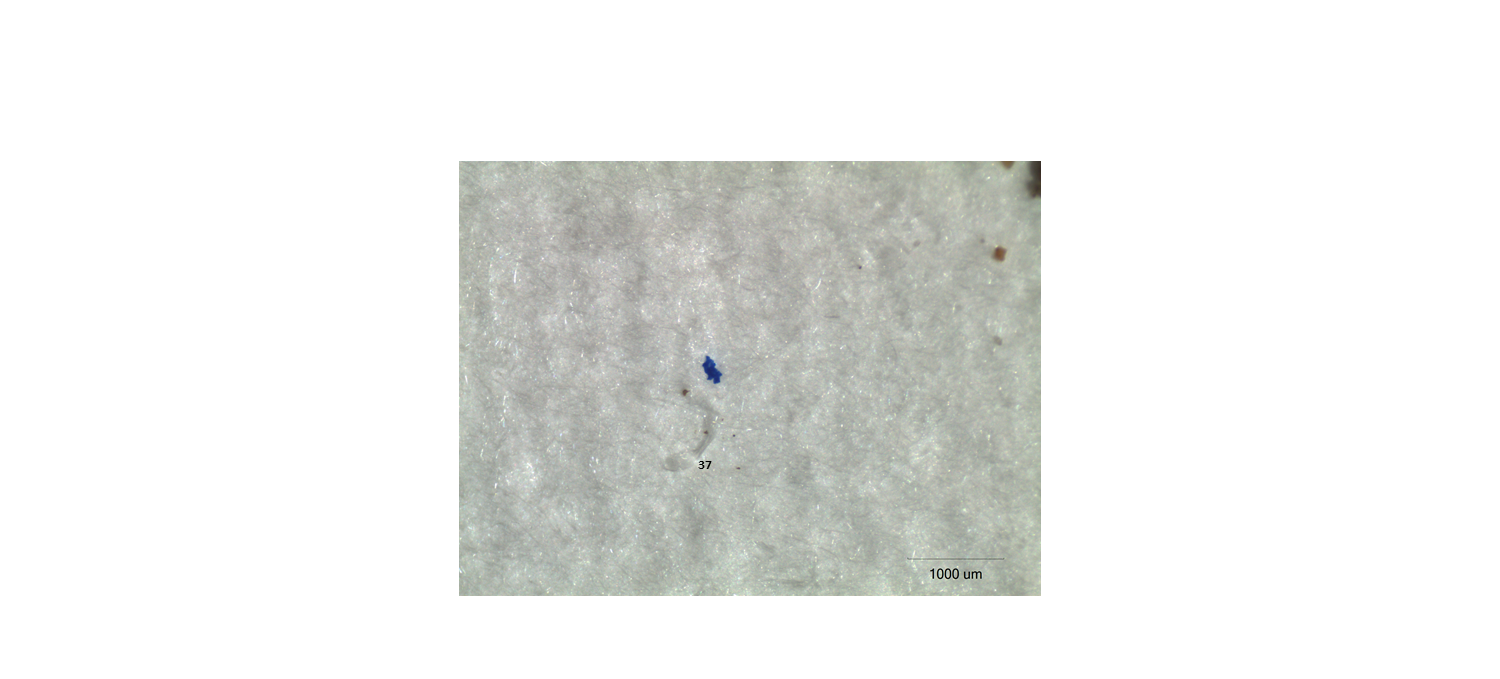 |

# Particle length

Particle length of the particles found in the sediment from the 5 different were relatively similar. The only significant differences were found between Ravn sø and Silkeborg Langsø (p<0.05, Wilcoxon’s test). Figure S8 illustrate the particle length at the different sites.


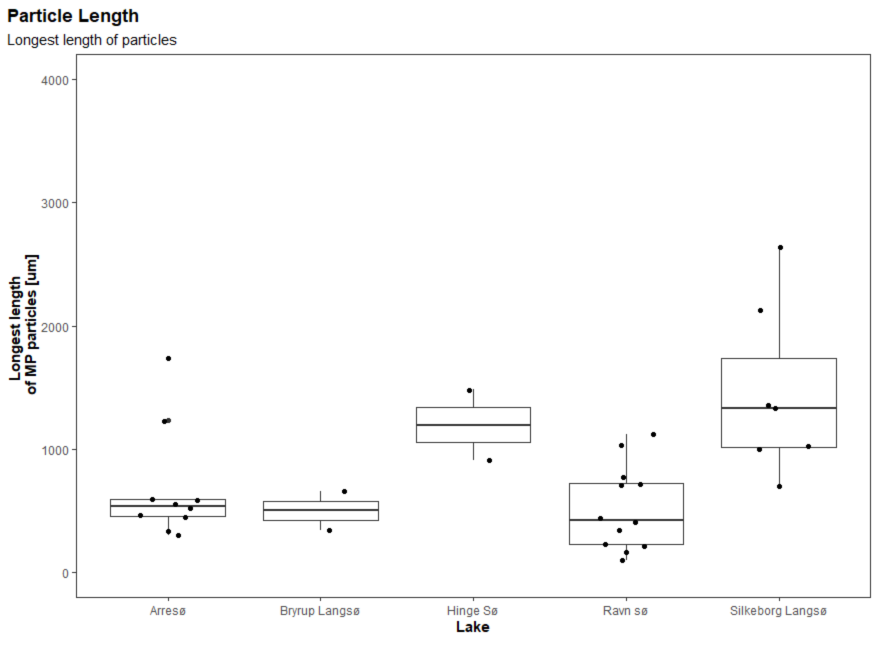


*Figure SI8. boxplot showing the size distribution of the microplastic found in the sediment samples from the 5 lakes (225 g sediment per site).*

# Principal component analysis

*
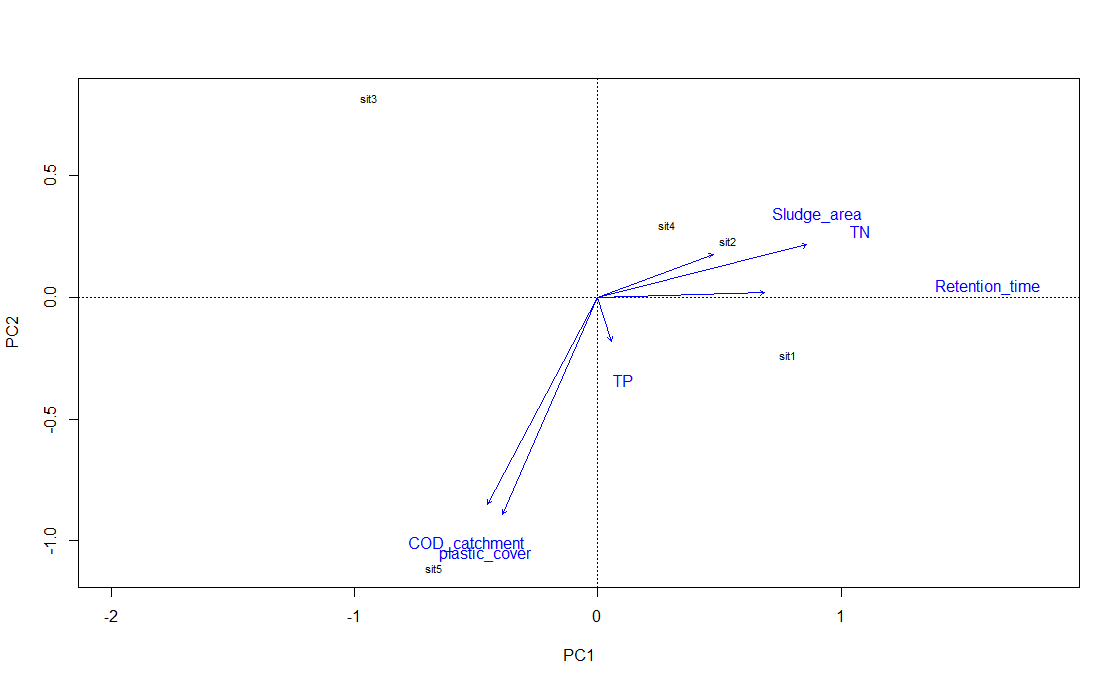
*

*Figure SI9. PCA plot illustrating the plastic types at the five sites (sit1-5) and how the parameters illustrated by arrows are contributing to each principal component. sit1=Arresø, sit2=Bryrup Langsø, sit3=Hinge sø, sit4=Ravn sø, sit5=Silkeborg Langsø*

*Table SI4: Results from the envfit analysis. The analysis shows that Prevalence of crops associated to the use of plastic cover (plastic cover) is the only slightly significant measure for describing the variance in the polymer type at the five different sites.*

|  | *PC1* | *PC2* | *r2* | *Pr(>r)* |
| --- | --- | --- | --- | --- |
| *TP* | *0.29144* | *-0.95659* | *0.0366* | *0.93333* |
| *TN* | *0.96954* | *0.24492* | *0.8209* | *0.14167* |
| *Plastic_cover* | *-0.40064* | *-0.91623* | *0.9931* | *0.04167 ** |
| *Retention_time* | *0.99960* | *0.02822* | *0.4911* | *0.46667* |
| *COD_catchment* | *-0.47014* | *-0.88259* | *0.9679* | *0.16667* |
| *COD_directly* | *-0.46967* | *-0.88284* | *0.9533* | *0.20000* |
| *Sludge_area* | *0.93587* | *0.35235* | *0.2679* | *0.63333* |

*Signif. codes: ‘***’ 0.001 ‘**’ 0.01 ‘*’ 0.05 ‘.’ 0.1 ‘ ’ 1*

*Table SI5: Explanation by each principal component. 83.6 % of the variance in data is described by the two first principal components.*

|  | *PC1* | *PC2* | *PC3* | *PC4* |
| --- | --- | --- | --- | --- |
| *Eigenvalue* | *3.0560* | *2.7941* | *1.0753* | *0.07462* |
| *Proportion Explained* | *0.4366* | *0.3992* | *0.1536* | *0.01066* |
| *Cumulative Proportion* | *0.4366* | *0.8357* | *0.9893* | *1.00000* |

# Particle found in mussel

*
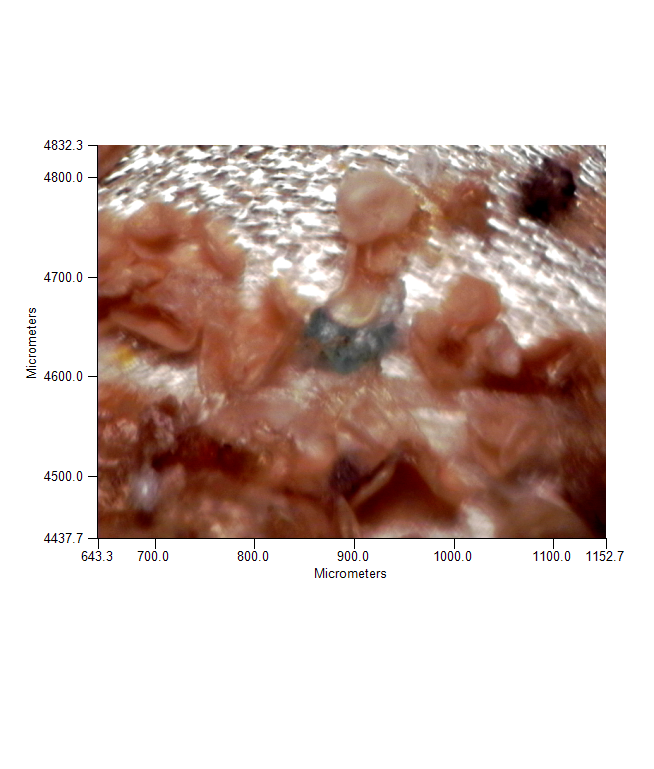
*

*Figure SI10: Blue PP particle that was found in the Dreissena polymorpha do not share the characteristics as the blue pp from the falcon tube lids in the blank.*

# Potential microplastic sources

## Discharge from point sources

When normalised for lake area, Silkeborg Langsø had by far the largest outflow of point sources in all three years both directly to the lake and to the catchment upstream (Figure 15 and Figure 16). The second highest outflow from point sources both directly to the lake and to the catchment was Bryrup Langsø, whilst the lake and lake catchment receiving the smallest input from point sources was Hinge sø. These point sources represent inputs covering wastewater effluent, industrial effluent, stormwater discharge and discharge from scattered dwellings, which could be suspected to contain microplastic particles. They are not confirmed microplastic point sources, but instead potential sources.


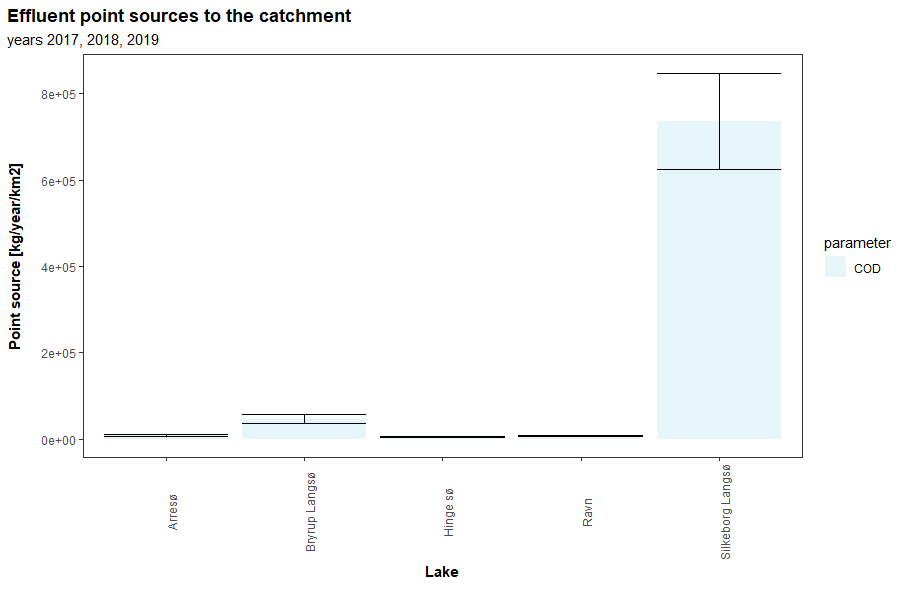


*Figure SI11: Discharge to catchment of the five lakes from point sources measured as biological oxygen demand over five days (BOD5), chemical oxygen demand (COD) and total discharge of water. Data is reported as annual discharge (kg) per lake area. Data can be seen below.*

*Table SI6: Point sources to the catchment of the 5 lakes in the years 2017, 2018, 2019, measured as chemical oxygen demand (COD) discharged to the catchment.*

| **Lake** | **Year** | **COD (kg/year/km2)** |
| --- | --- | --- |
| Arresø | 2017 | 12013.47 |
| Bryrup Langsø | 2017 | 50397.29 |
| Hinge sø | 2017 | 4979.659 |
| Ravn | 2017 | 6654.2 |
| Silkeborg Langsø | 2017 | 799015.6 |
| Arresø | 2018 | 6492.964 |
| Bryrup Langsø | 2018 | 35765.36 |
| Hinge sø | 2018 | 4008.766 |
| Ravn | 2018 | 5736.517 |
| Silkeborg Langsø | 2018 | 607457.6 |
| Arresø | 2019 | 8255.055 |
| Bryrup Langsø | 2019 | 54376.32 |
| Hinge sø | 2019 | 5741.935 |
| Ravn | 2019 | 7860.674 |
| Silkeborg Langsø | 2019 | 801859.8 |


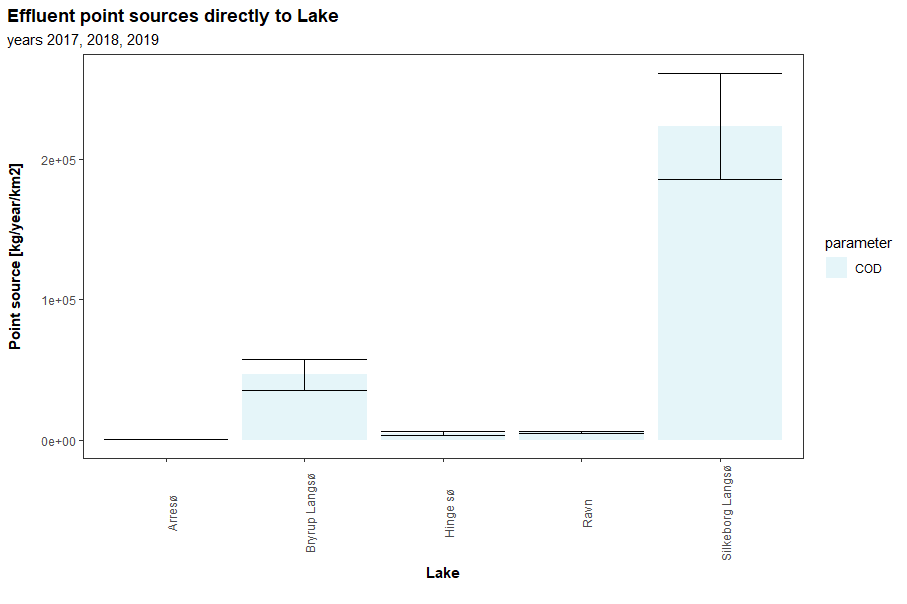


*Figure SI12: Discharge directly into the lake from point sources measured as chemical oxygen demand (COD). Data is reported as annual discharge (kg) per lake area. Data can be seen below.*

*Table SI7: Point sources directly to each of the 5 lakes in the years 2017, 2018, 2019, measured as chemical oxygen demand (COD) to the catchment.*

| **Lake** | **Year** | **COD (kg/year/km2)** |
| --- | --- | --- |
| Arresø | 2017 | 265.9797 |
| Bryrup Langsø | 2017 | 50960.53 |
| Hinge sø | 2017 | 4995.235 |
| Ravn | 2017 | 6416.11 |
| Silkeborg Langsø | 2017 | 221287.4 |
| Arresø | 2018 | 617.3748 |
| Bryrup Langsø | 2018 | 34254.92 |
| Hinge sø | 2018 | 3233.74 |
| Ravn | 2018 | 4655.859 |
| Silkeborg Langsø | 2018 | 186445.7 |
| Arresø | 2019 | 870.8303 |
| Bryrup Langsø | 2019 | 54984.03 |
| Hinge sø | 2019 | 5759.896 |
| Ravn | 2019 | 5362.652 |
| Silkeborg Langsø | 2019 | 262340.2 |

## Agricultural use:

The main crops across all five catchments were grass, barley, wheat, maize, rapeseed and sugar beet. None of these use plastic mulching, but may be subject to sludge application to land. Grass production may involve the use of silage films, which are used extensively in Denmark (Briassoulis et al., 2013). When considering the crops which may be subject to agricultural plastic use (i.e. fruit, vegetables, potatoes), the area accounted for by these varied between the catchments. Silkeborg Langsø had the largest area (approximately 800 ha), whilst Hinge sø had no fields with these crop types (see Supplementary material Figure 17 & Table 4).

Regarding the application of sewage sludge to land, the volume varied greatly among the lakes and between the years (Table 3). Corrected for lake area, Bryrup Langsø catchment has the highest total sludge application, whilst Hinge sø was the lowest.

*Table SI8: Sludge application in the catchment of the 5 lakes. Given as an average volume of the years 2017, 2018, 2019 in Tons/km2.*

| Lake | Average sludge application for each catchment pr lake area (Tons/km2) | sd |
| --- | --- | --- |
| Arresø | 98.8 | 44.15 |
| Bryrup Langsø | 1315.8 | 3721.62 |
| Hinge sø | 26.9 | 76.03 |
| Ravn | 461.8 | 191.76 |
| Silkeborg Langsø | 152.4 | 114.22 |


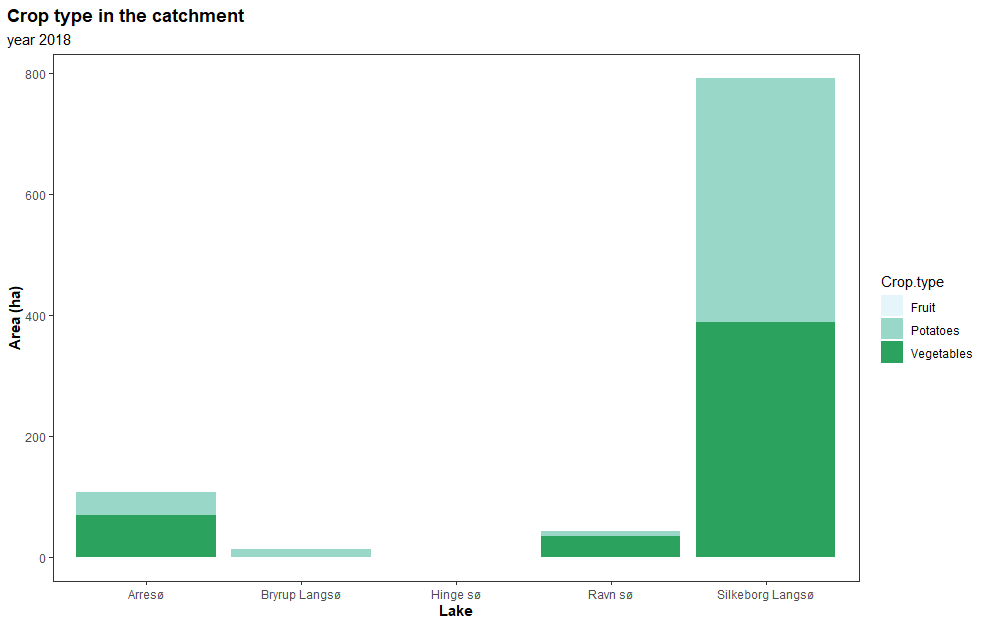


*Figure SI13. Area (ha) of the different crop types per catchment of the five lakes that is normally associated to the use of plastic cover.*

*Table SI9: Main crop types in the catchment and area (ha) of the different crop types per catchment of the five lakes that is normally associated to the use of plastic cover.*

| **Lake** | **Main crop types** | **Potatoes (ha)** | **Vegetables (ha)** | **Fruit (ha)** |
| --- | --- | --- | --- | --- |
| Arresø | barley, grass, wheat, rapeseed, sugarbeat, maize | 38 | 69.9 | 0 |
| Bryrup Langsø | Wheat, grass, maize, sugarbeat, barley, rapeseed | 13.6 | 0 | 0 |
| Hinge sø | Grass, barley, wheat, maize | 0 | 0 | 0 |
| Ravn sø | Grass, wheat, maize, barley | 8.3 | 35.6 | 0 |
| Silkeborg Langsø | Grass, barley, wheat, maize | 402 | 388.4 | 0 |

## In-Lake bio/chemical data

When comparing key environmental variables, the five lakes varied somewhat in their concentrations. Both Total N and P showed relative high concentrations for all lakes. Total P were relatively similar for all lakes. Hinge Sø and silkeborg Langsø has the lowest TN concentrations. These data illustrates, that we have lakes that are affected by catchment processes e.g. fertilizing that results in high TN concentrations and discharge from WWTP that adds TP.


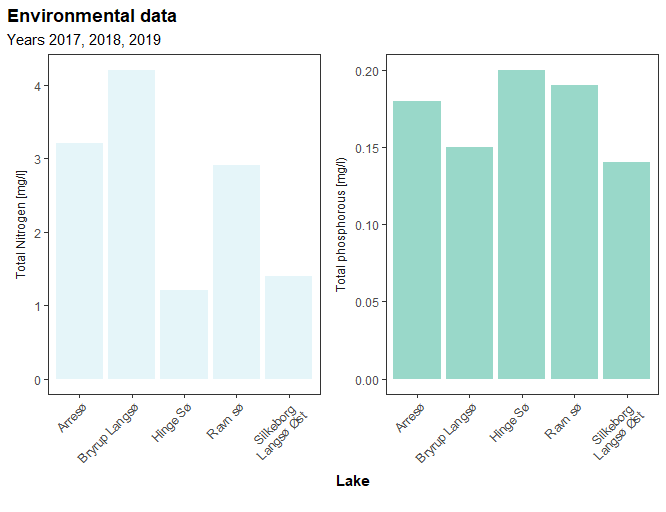


*Figure SI14: Graphs illustrating concentrations of Total Nitrogen and Total phosphorous in the five lake.*

*Table SI10: in situ studies of microplastic in freshwater bivalves, species, size of found microplastic and detection size of the study are listed.*

| **Reference** | **Species** | **Size of microplastic taken up** | **Detection size** |
| --- | --- | --- | --- |
| (Su et al. 2016) | Corbicula fluminea (Asian Clam) | 5-5000 | 5-5000 |
| (Su et al. 2018) | Corbicula fluminea (Asian Clam) | 21-4830 | 5-5000 |
| (Berglund et al. 2019) | Anodonta anatina | No information | <5000 |
| (Domogalla-Urbansky et al. 2019) | Unio pictorum | 8-183 | 5-5000 |
| (Schessl et al. 2019) | Dreissena polymorpha | No particles found | 50-5000 |
| (Schessl et al. 2019) | Dreissena bugensis | No particles found | 50-5000 |
| (Baldwin et al. 2020) | Dreissena bugensis | No information | 125-5600 |
| (Baldwin et al. 2020) | Corbicula fluminea (Asian Clam) | No information | 125-5600 |
| (Pazos et al. 2020) | Limnoperna fortunei | <100-5000 | <5000 |
| (Wardlaw &Prosser 2020) | Lasmigona costata | 21 - 298 | No information |
| (Doucet et al. 2021) | Margaritifera margaritifera L. | Not measured | <5000 |
| (Pastorino et al. 2021) | Driessena polymorpha | 149.1-2289.17 | 10-5000 |
| (Hoellein et al. 2021) | Driessena polymorpha | Not measured |  |
